# Manipulation of prostate cancer metastasis by locus-specific modification of the CRMP4 promoter region using chimeric TALE DNA methyltransferase and demethylase

**DOI:** 10.18632/oncotarget.3192

**Published:** 2015-04-09

**Authors:** Ke Li, Jun Pang, Huaiyan Cheng, Wei-Peng Liu, Jin-Ming Di, Heng-Jun Xiao, Yun Luo, Hao Zhang, Wen-Tao Huang, Ming-Kun Chen, Liao-Yuan Li, Chun-Kui Shao, Ying-Hong Feng, Xin Gao

**Affiliations:** ^1^ Department of Urology, the Third Affiliated Hospital, Sun Yat-Sen University, Guangzhou 510630, China; ^2^ Department of Pharmacology, Uniformed Services University of the Health Sciences, Bethesda MD20814, USA; ^3^ Department of Urology, the First Affiliated Hospital of Nan Chang University, Nanchang 330006, China; ^4^ Department of Pathology, the Third Affiliated Hospital, Sun Yat-Sen University, Guangzhou 510630, China

**Keywords:** prostate cancer, metastasis, transcription activator-like effectors (TALEs), CRMP4, epigenetic manipulation

## Abstract

Prostate cancer is the most commonly diagnosed non-cutaneous cancer and one of the leading causes of cancer death for North American men. Whereas localized prostate cancer can be cured, there is currently no cure for metastatic prostate cancer. Here we report a novel approach that utilizes designed chimeric transcription activator-like effectors (dTALEs) to control prostate cancer metastasis. Transfection of dTALEs of DNA methyltransferase or demethylase induced artificial, yet active locus-specific CpG and subsequent histone modifications. These manipulations markedly altered expression of endogenous CRMP4, a metastasis suppressor gene. Remarkably, locus-specific CpG demethylation of the CRMP4 promoter in metastatic PC3 cells abolished metastasis, whereas locus-specific CpG methylation of the promoter in non-metastatic 22Rv1 cells induced metastasis. CRMP4-mediated metastasis suppression was found to require activation of Akt/Rac1 signaling and down-regulation of MMP-9 expression. This proof-of-concept study with dTALEs for locus-specific epigenomic manipulation validates the selected CpG methylation of CRMP4 gene as an independent biomarker for diagnosis and prognosis of prostate cancer metastasis and opens up a novel avenue for mechanistic research on cancer biology.

## INTRODUCTION

Men with metastatic prostate cancer, are faced with poor prognosis, having median survival times in the range of only 3–7 years [1]. Although androgen ablation, currently the treatment of choice for metastatic prostate cancer, can lead to remissions, tumors frequently return in a “castration-resistant” form (i.e. castration-resistant prostate cancer, CRPC). The current standard care for treating CRPC is systemic, docetaxel-based chemotherapy, increasing the overall survival of patients by about 2 months compared to the previous standard “mitoxantrone plus prednisone” regimen [2, 3]. Recently sipuleucel-T [4], cabazitaxel [5], abiraterone (Zytiga) [6], and MDV3100 [7] have shown more prolonged overall survival benefit and have been approved by the FDA for treatment of the disease. However, none of these drugs are curative; they only marginally improve patients' overall survival. Clearly, establishment of more effective therapeutic targets and drugs specifically aimed at mCRPC is of critical importance for improved disease management and patient survival [8]. Similarly, there is a dire need for improved prognostic metastatic biomarkers to determine whether primary prostate cancers are potentially aggressive or indolent; in the latter case, patients can be spared from over treatment [9].

Metastasis suppressor genes (MSGs) are negative regulators of metastasis [10, 11]. Compelling evidence indicates that modifications of DNA methylation and histones can silence the expression of MSGs, leading to the development of metastasis [12–14]. This notion is supported by studies of CRMP4, a novel prostate cancer MSG recently identified by our laboratory [15]. Conversely, reactivation of MSGs has the potential to inhibit cancer metastasis and may be useful as a co-treatment of micro-metastases present in localized, hormone-refractory prostate cancers undergoing radiation therapy [16, 17].

At present, most epigenomic modifications in laboratory research and clinical settings are achieved with non-specific approaches such as use of the HDAC inhibitor valproic acid and methyltransferase inhibitors 5-azacytidine and 5-aza-2-deoxycytidine (decitabine) [18–20]. Although site-selective methylation with chimeric DNA methyltransferases has been achieved using DNA-binding domains derived from various native DNA-binding proteins [21] and artificial zinc finger DNA-binding domains [22], these modes of methylation suffer from poor specificity.

Recent breakthroughs in demystifying transcription activator-like effectors (TALEs) for DNA recognition with high specificity [23, 24] has made locus-specific targeting possible for a wide variety of downstream applications [25]. Moreover, the recent discovery of DNA demethylase (TET1) [26, 27], and successful reactivation of endogenous gene expression with such a demethylase guided by TALE has opened up an exciting avenue for locus-specific modification of genes [28].

We envisaged that TALE-assisted locus-specific modifications of the CRMP4 promoter region could alter expression of the gene, and thus control prostate cancer metastasis. In the present study, we demonstrate that designed chimeric TALEs (dTALEs) containing a catalytic domain of DNA methyltransferase DNMT3A or DNA demethylase TET1 can turn prostate cancer metastasis on or off by altering CRMP4 expression through locus-specific modification of the gene promoter.

## RESULTS

### CRMP4 promoter region and its sensitivity to regulation of CpG modification

**Figure 1:**
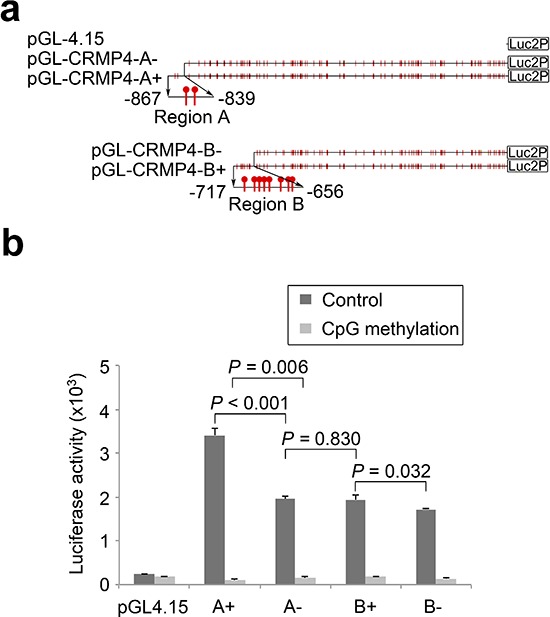
Regulation of CRMP4 promoter activity by CpG modification **(a)** Illustration of the four CRMP4 promoter-driven luciferase reporters designated as A+ (−867/+114), A− (−839/+114), B+ (−717/+114), and B− (−656/+114). **(b)** Luciferase activities of the four CRMP4 promoter reporters that were pre-treated with or without M.SssI. One-way ANOVA was used to analyze the difference among the four groups luciferase reporters designated, and the differences between groups determined by the Student's *t*-test were considered to be significant at a *P* value less than 0.05/3 after correction. The error bars in **b** are s.e.m.

We previously reported that CpG methylation in CRMP4 promoter Regions A and B (Supplementary Figure S1a) correlated with the metastatic status of prostate cancer [15]. To determine whether CRMP4 expression is sensitive to regulation of CpG modification and investigate if CpG methylation represses transcription of the gene, the predicted CRMP4 core promoter (Supplementary Figure S1b) was utilized to construct four luciferase reporters (Figure 1a). As shown in Figure 1b, all four CRMP4 promoter regions were able to drive substantial luciferase expression. Inclusion of Region A (A+) further increased reporter activity by 42.7%, whereas inclusion of Region B (B+) only increased reporter activity by 11.2%, suggesting that Region A plays a greater enhancer role. Importantly, considerable luciferase expression observed in reporter B− (50.5% of the reporter A+ activity) suggests that the predicted core promoter and the initial exon play a pivotal role in driving CRMP4 expression. Reporter A−showed little difference over reporter B+ in luciferase activities, suggesting that the 122 bp sequence (−839 to −717) between Regions A and B have little effect on CRMP4 expression.

CpG methylation in a promoter region represses transcription of the gene in most cases, but not in all cases [29]. Here, Figure 1b shows that treatment with M.SssI induced considerable repression of luciferase expression in all four reporters, suggesting that the CRMP4 promoter region is highly sensitive to CpG methylation. In the presence of M.SssI treatment, inclusion of Region A in reporter A+ further reduced luciferase activity (34%) to almost extinction (Supplementary Figure S1c). Given the fact that Region A further increased luciferase activity in the absence of M.SssI treatment, this result suggests that Region A may act as a CpG methylation-sensitive repressive enhancer. As compared to reporter B+, reporter A− and B− showed little difference in luciferase activity, further suggesting that the 122 bp sequence does not affect CRMP4 expression regardless of the methylation status, and that Region B is a CpG methylation-insensitive enhancer.

### Generation of dTALEs for locus-selective CpG modification

**Figure 2:**
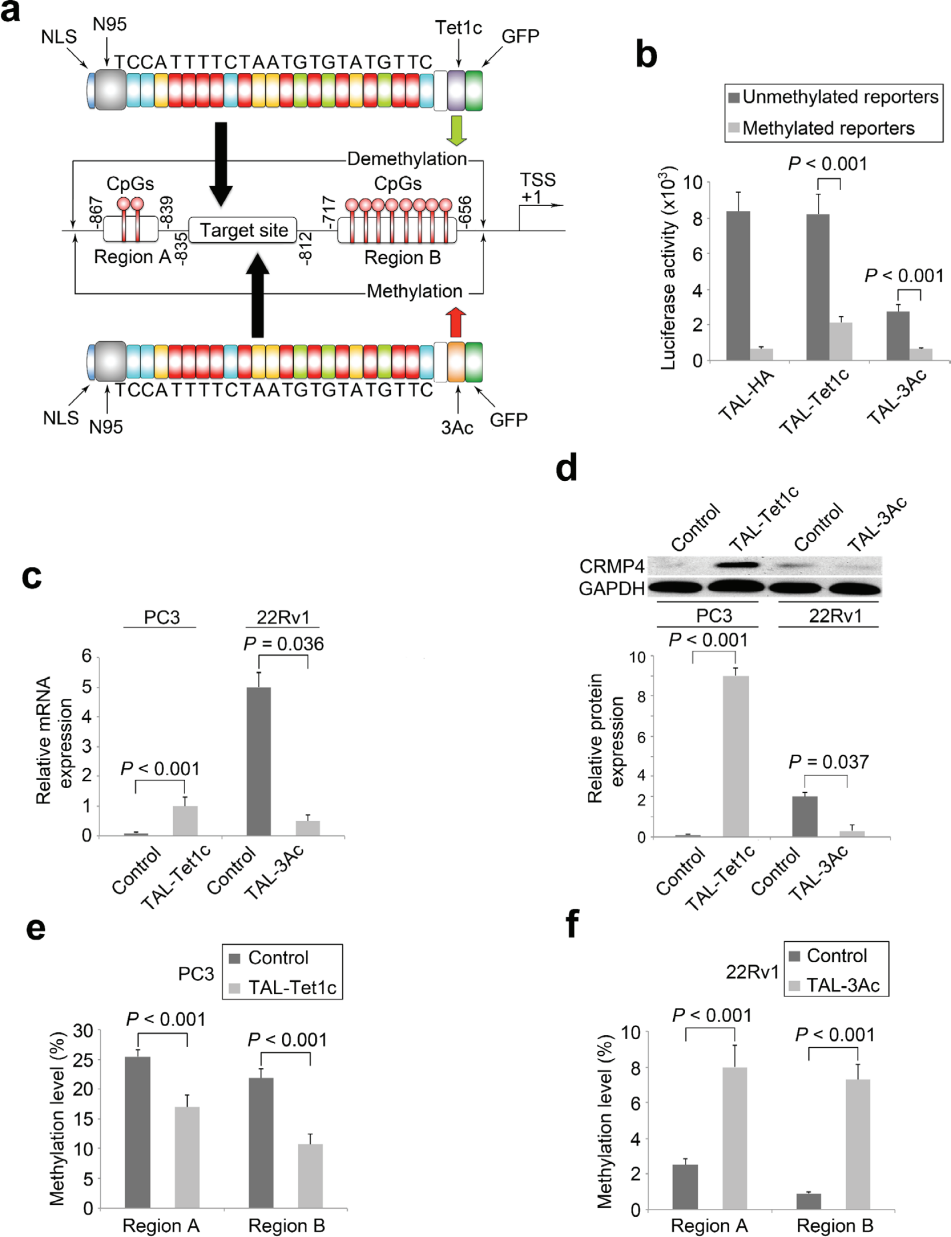
Locus-specific modulation of CRMP4 expression by dTALEs **(a)** Illustration of the dTALEs. The synthetic TALE DNA-binding domain, the 23 bp targeting sequence from CRMP4 promoter region, nuclear localization signal (NLS), the truncated N-terminal domain (N95), the catalytic domain of Tet1 (Tet1c), the catalytic domain of DNMT3A (3Ac), and the other functional domain such as GFP are shown. The CRMP4 promoter structure (middle panel of Figure 2a) is drawn on a non-proportional scale. TSS: translation start site. **(b)** Luciferase activities altered by dTALEs through locus-specific CpG modification. Co-transfections with dTALEs and the CpG-free CRMP4-pCpGL reporter pre-treated with and without M.SssI were performed in HEK293 and COS-1 cells. **(c)** Alteration of endogenous CRMP4 mRNA expression in prostate cancer cells detected using qRT-PCR. The PC3 and 22Rv1 cells were transfected with CRMP4-TAL-3Ac, CRMP4-TAL-Tet1c, and empty phCMV1 vector to induce locus-specific CpG modifications. **(d)** Alteration of endogenous CRMP4 protein expression in prostate cancer cells detected using Western blotting. The prostate cancer cells were treated as described for Figure 3c. **(e)** CpG methylation frequencies of CRMP4 promoter Region A and Region B detected in the PC3 cells using pyrosequencing. The PC3 cells were transfected with CRMP4-TAL-Tet1c or empty phCMV1 vector as control. **(f)** CpG methylation frequencies of CRMP4 promoter Region A and Region B detected in the 22Rv1 cells using pyrosequencing. The 22Rv1 cells were transfected with CRMP4-TAL-3Ac or empty phCMV1 vector as control. The *P* values in **b–f** were determined with the Student's *t*-test. The error bars in **b–f** are s.e.m.

To achieve locus-specific CpG modification within the pre-determined CRMP4 promoter region, a short sequence between Regions A and B was selected as a TALE targeting motif (23 bp, Figure 2a, Supplementary Figures S1, S2a). Query of this 23 bp sequence against the human genome in Blast search revealed no identical sequence stretches that are long enough to become a concern for off-target binding (Supplementary Figure S1d).

To verify DNA binding specificity and potency of the TALE, co-transfection of CRMP4-TAL-vp64 and CRMP4-Luc2pin HEK293 cells induced a 21-fold increase in luciferase activity over control background (Supplementary Figures S2b–S2c), strongly suggesting that the synthetic TALE DNA binding domain can specifically recognize the selected targeting motif with desired potency.

To introduce epigenomic modifications specifically to the predetermined CRMP4 promoter region, CRMP4-TAL-3Ac and CRMP4-pCpGL reporter (Figure 2b) were transfected into COS1 cells, inducing a 67.5% reduction in luciferase activity. Interestingly, co-transfection of CRMP4-TAL-Tet1c and M.SssI-pretreated CRMP4-pCpGL reporter rendered a significant increase in luciferase activity by 3.3-fold (Figure 2b). These results suggest that these dTALEs can selectively modify the CpG methylation within the selected the CRMP4 promoter region, leading to up and down regulation of expression of the CRMP4 gene.

### Modulation of CRMP4 expression in prostate cancer cells by locus-specific CpG modifications

To determine whether CRMP4-TAL-Tet1c and CRMP4-TAL-3Ac are able to induce locus-specific CpG modifications of endogenous CRMP4 and consequently alter the expression of the gene, these plasmids were transfected into metastatic PC3 cells that express little CRMP4 and into non-metastatic 22Rv1 cells expressing abundant CRMP4, respectively (Figures 2c, 2d). In agreement with the results from the above mentioned reporter assays, CRMP4 expression in PC3 cells expressing CRMP4-TAL-Tet1c and 22Rv1 cells expressing CRMP4-TAL-3Ac showed significant up and down-regulation, respectively, in both mRNA (Figure 2c) and protein (Figure 2d) levels relative to their specific controls.

To determine whether and to what frequency and range the targeted CpG modifications did take place in the CRMP4 promoter region, the genomic DNA samples from the PC3 and the 22Rv1 cells expressing CRMP4-TAL-Tet1c and CRMP4-TAL-3Ac, respectively, were extracted and pre-treated with bisulfite for pyrosequencing. Figures 2e–2f show that ectopic expression of CRMP4-TAL-Tet1c in PC3 cells decreased the average methylation frequencies from 25.5% to 17.0% in Region A and from 21.9% to 10.7% in Region B, relative to the controls (Figure 2e, Supplementary Figures S3a–S3b). In contrast, ectopic expression of CRMP4-TAL-3Ac increased the average methylation frequencies from 2.5% to 8.0% in Region A and from 0.9% to 7.3% in Region B, relative to the controls (Figure 2f, Supplementary Figures S4a–S4b).

Further sequencing toward 5′ of Region A and 3′ of Region B identified that dTALE-mediated CpG modifications extended up to 300 bp in both directions from the TALE-targeting sequence, suggesting epigenomic modifications outside of Regions A and B also occurred for both DNA demethylase (Supplementary Figures S3c–S3f) and methyltransferase (Supplementary Figures S4c–S4f). In contrast, CpG methylation levels remained unchanged for the selected genes *RASSF1A*, *p16* and *TWIST1* in the PC3 and the 22Rv1 cells with or without transfection of dTALEs, suggesting negligible off-target modifications (Supplementary Figures S5a–S5c).

### Induction of locus-specific histone modification by locus-specific CpG modification

**Figure 3:**
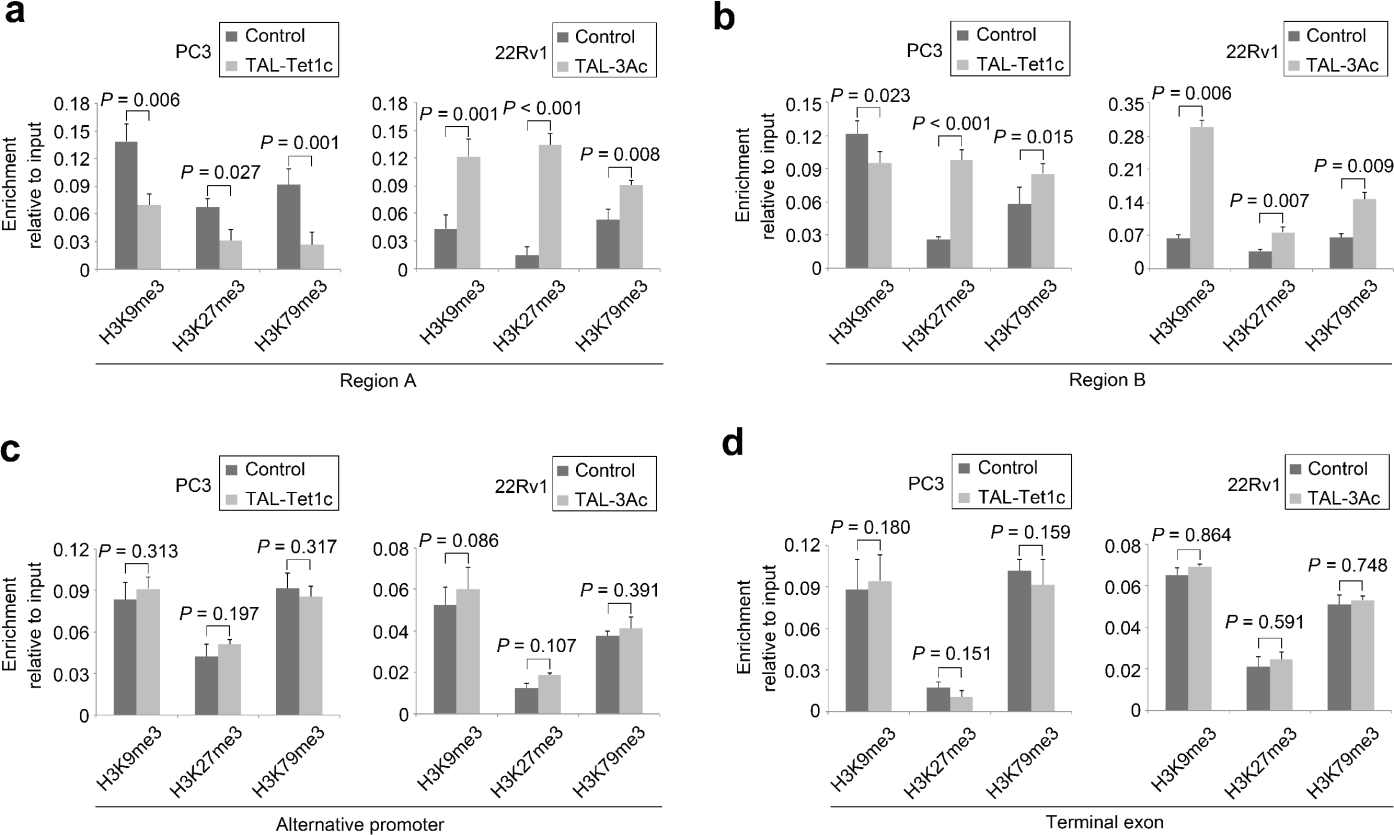
Histone modifications in the PCa cells expressing dTALEs Ectopic expression of CRMP4-TAL-Tet1c in PC3 cells and CRMP4-TAL-3Ac in 22Rv1 cells induced the histone modifications at H3K9me3, H3K27me3 and H3K79me3 in **(a)** CRMP4 promoter Region A (−867/−839), **(b)** CRMP4 promoter Region B (−717/−656), **(c)** CRMP4 alternative promoter located 56 kb to the 5′ of the start codon, **(d)** CRMP4 terminal exon located 60 kb to the 3′ of the start codon. Empty phCMV1 vector was transfected in PC3 and 22Rv1 cells as controls. CRMP4 gene is located in chromosome 5. The *P* values in **a–d** were determined with the Student's *t*-test. The error bars in **a–d** are s.e.m.

To determine whether the artificial CpG modifications induced histone modifications at the same or nearby loci of the CRMP4 promoter region, chromatin immunoprecipitation (ChIP) assays were performed to detect H3K9me3, H3K27me3 and H3K79me3. Figures 3a–3b show that despite the ectopic expression, Regions A and B of 22Rv1 cells, but not of PC3 cells, had identical magnitudes of histone modifications at all three sites, suggesting that the Regions A and B of the CRMP4 promoter employ the same nucleosome in 22Rv1 cells, but different nucleosomes in PC3 cells. These findings also suggest that CRMP4 promoter sequences involved in nucleosome formation might be different in PC3 and 22Rv1 cells. More importantly, Figures 3a–3b also show significant decreases of the trimethylation in Regions A and B in PC3, but dramatic increases in 22Rv1 cells. Of particular note is Region A at which the trimethylations were modified most, supporting its major role in regulating the expression of the gene. Contradictorily, ectopic expression of CRMP4-TAL-Tet1c in PC3 cells increased secondary trimethylations at H3K27 and H3K79 (Figure 3b, left panel).

Ectopic expression of CRMP4-TAL-Tet1c in PC3 cells and CRMP4-TAL-3Ac in 22Rv1 cells did not alter the H3K9me3, H3K27me3, and H3K79me3 status in the CRMP4 alternative promoter and terminal exon (Figures 3c–3d). Consistently, the trimethylation status at the selected H3K sites also remained unchanged for control genes *GAPDH*, *RASSF1A*, and *p16* (Supplementary Figures S6a–S6c) when CRMP4-TAL-Tet1c and CRMP4-TAL-3Ac were expressed. These negative results suggest that the locus-specific epigenomic modifications occur only within the localized target region without alteration of the distal regions of the epigenome.

### Manipulation of prostate cancer cell metastasis by dTALEs

**Figure 4:**
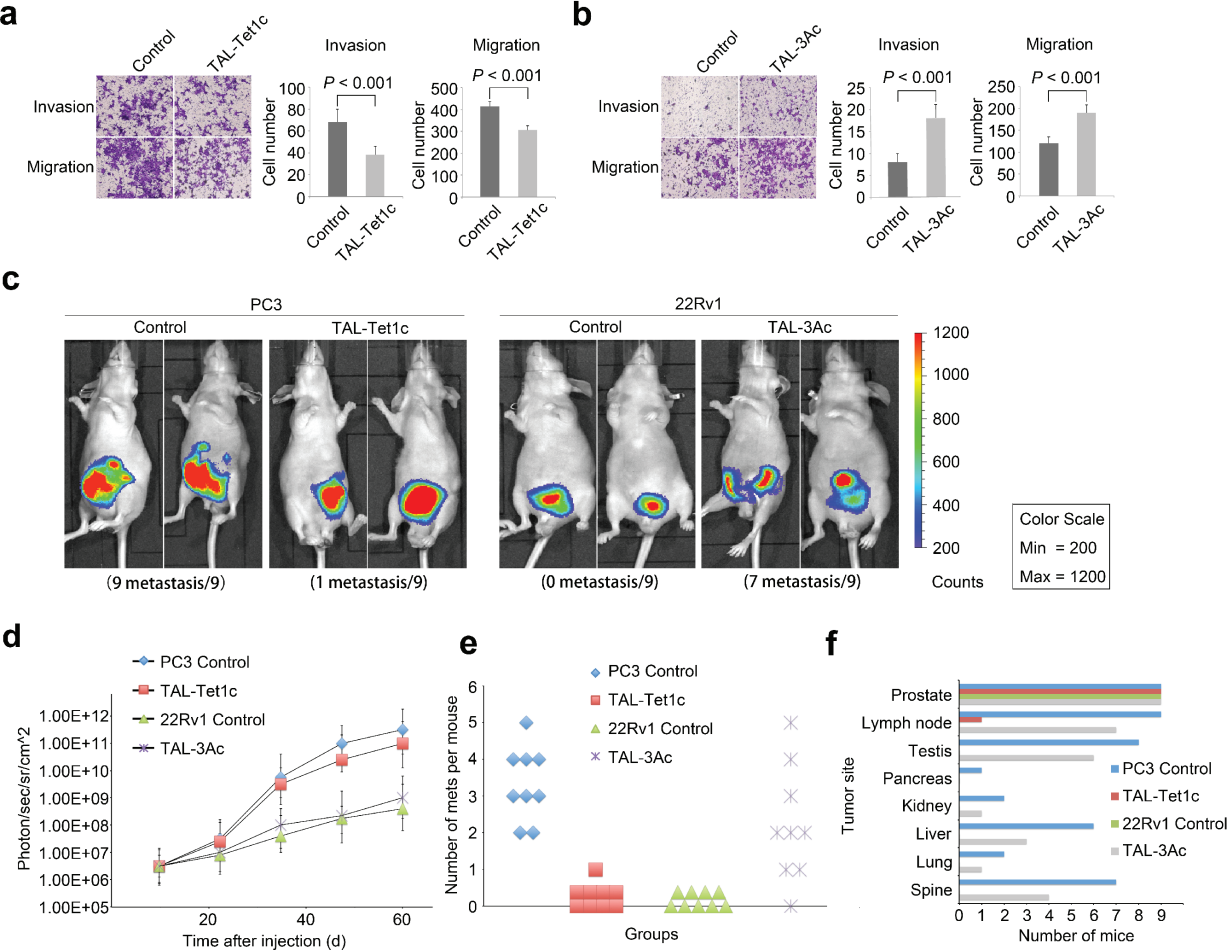
*In vitro* and *in vivo* manipulation of prostate cancer cell metastasis by dTALEs *In vitro* migration and tissue invasion of the PC3 cells **(a)** and the 22Rv1 cells **(b)** transfected with specified dTALEs or empty phCMV1 vector as control. Left panel: representative images; Right panel: results in means of three independent experiments. Significance was determined with the Student's *t*-test. **(c)** Xenogen images of mice with orthotopic implantation of the dTALE-expressing PC3 and 22Rv1 cells infected with luciferase-expression lentivirus LP-RLUC-LV. **(d)** Tumor volume of PC3 and 22Rv1 cells with and without expression of the specified dTALEs or empty phCMV1 vector as control. The volume was calculated from the Xenogen images. **(e)** Dot plot depicting number of metastases per mouse in animals injected with PC3 or 22Rv1 cells expressing specified dTALEs or empty phCMV1 vector as control. **(f)** Organ distribution frequency of tumor metastasis. All mice were autopsied and the organs were measured by detecting luciferase activity, respectively. The *P* values in **a, b** were determined with the Student's *t*-test. The error bars in **a, b, d** are s.e.m.

To determine whether the locus-specific epigenomic modifications are necessary and sufficient to transform tumor progression *in vitro* and *in vivo*, the metastatic PC3 cells expressing CRMP4-TAL-Tet1c and the non-metastatic 22Rv1 cells expressing CRMP4-TAL-3Ac were examined for tissue invasive and cell migratory activities. Figure 4a shows that CRMP4-TAL-Tet1c significantly reduced tissue invasion and migration of PC3 cells. Figure 4b shows that CRMP4-TAL-3Ac induced significant tissue invasion and migration of 22Rv1 cells.

To facilitate *in vivo* evaluation of cancer progression, PC3 and 22Rv1 cells co-infected with luciferase reporter and dTALEs were injected into prostates of mice, respectively. As shown in Figure 4c, all the mice (*n* = 9) injected with control PC3 cells developed metastases, whereas 8 out of 9 animals (88.9%) injected with PC3 cells expressing CRMP4-TAL-Tet1c did not. Importantly, while the tumors of the latter were relatively smaller, 7 out of 9 mice (77.8%) harboring CRMP4-TAL-3Ac-infected 22Rv1 cells exhibited partially promoted metastasis (Figure 4c), whereas none of the control mice (*n* = 9) did (Figure 4c). These loss-of-function and gain-of-function results with both PC3 and 22Rv1 cells strongly indicate that CRMP4 expression is not only necessary, but also sufficient for termination of prostate cancer metastasis.

Although the PC3-driven tumors presented with higher photon flux than the 22Rv1-driven tumors, there were no significant photon differences between the PC3-driven subgroups of primary tumors. This was also true for 22Rv1-driven subgroups (Figure 4d). These results imply that CRMP4 has little effect on primary tumor development, but specifically and significantly mediates metastasis.

Prior studies have shown that metastatic prostate cancer in patients displayed heterogeneity in organ distribution, including lung, liver, kidneys and bone [30, 31]. This study shows that, CRMP4-TAL-Tet1c significantly reduced the PC3 cell-induced metastatic lesions in multiple organs including, most frequently, proximal and distal lymph nodes followed by testis, spine, liver, lung, kidney and pancreas. Interestingly, CRMP4-TAL-3Ac significantly increased the metastatic tumor distributions (Figures 4e–4f).

### Identification of Akt-Rac1-MMP9 signaling in CRMP4-mediated metastasis

**Figure 5:**
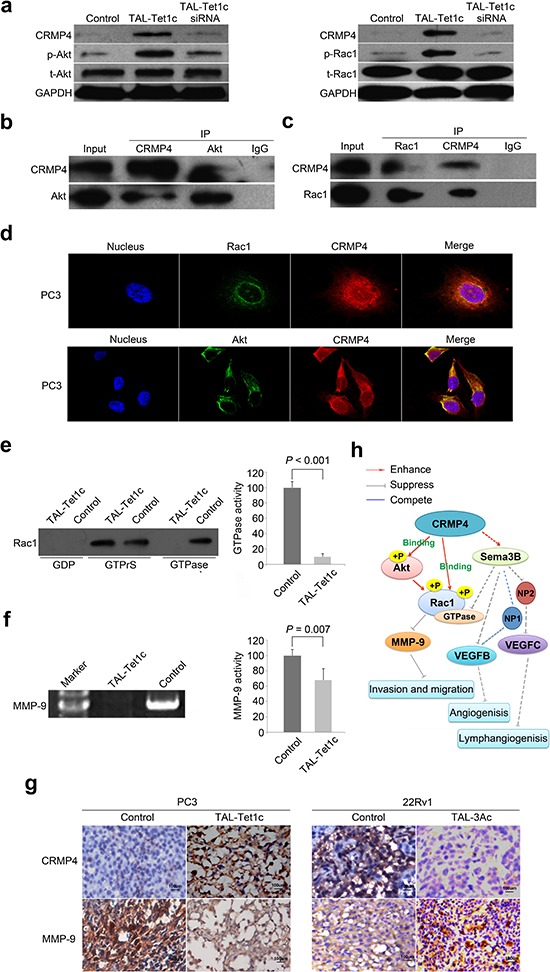
Akt-Rac1-MMP9 signaling pathway in CRMP4-mediated suppression of metastasis **(a)** Western blot detection of phosphorylation and expression of Akt and Rac1 in the PC3 cells transfected with CRMP4-TAL-Tet1c or empty vector phCMV1 as control. CRMP4 siRNA was utilized to verify the loss-of-function. **(b)** Akt and CRMP4 interaction detected in the PC3 cells with differential Co-IP and Western blot. **(c)** Rac1 and CRMP4 interaction detected in the PC3 cells with differential Co-IP and Western blot. **(d)** Subcellular co-localization of CRMP4 with Akt and Rac1, respectively, in the CRMP4-TAL-Tet1c-expressing PC3 cells detected using confocal images. **(e)** Rac1 GTPase activity detected in the CRMP4-TAL-Tet1c-expressing PC3 cells using a Pull-down assay. **(f)** MMP-9 activity detected in the CRMP4-TAL-Tet1c-expressing PC3 cells using a Gelatin zymography assay. **(g)** IHC detection of opposite expression of CRMP4 and MMP-9 in the primary tumors from mice injected with PC3 or 22Rv1 cells expressing specified dTALEs. **(h)** Schematic of CRMP4-mediated signaling pathway involving phosphorylation of Akt and Rac1, activity of Rac1 GTPase and MMP-9, and repressed expression of MMP-9, VEGFB and VEGFC^15^, collectively leading to suppression of prostate cancer metastasis (Solid-lines: findings of this manuscript. Dashed-lines: previously published data and data not shown). The *P* values in **e** and **f** were determined with the Student's *t*-test. The error bars in **e** and **f** are s.e.m.

The mechanisms underlying CRMP4-mediated metastasis suppression remain largely unknown. Samples prepared from PC3 cells expressing CRMP4-TAL-Tet1c were arrayed on 1318 tumor-associated protein targets with their site-specific phospho-antibodies. The antibody microarray revealed that CRMP4-TAL-Tet1c significantly activated the phosphorylation status for a variety of targeted signaling proteins (Supplementary S7a and Supplementary Table S1), in particular Akt and Rac1. Interestingly, Akt and Rac1 expressions were not altered as shown in qRT-PCR (Supplementary Figure S7b) and Western blot assays (Figure 5a). Consistently, Figure 5a shows that CRMP4-TAL-Tet1c did significantly elevate the phosphorylation of Akt Ser473 and Rac1 Ser71. Such elevation was subsequently blocked by siRNA that repressed the CRMP4 expression (Figure 5a).

To understand how CRMP4 induces Akt and Rac1 phosphorylation, a co-immunoprecipitation (Co-IP) experiment was performed. Figure 5b–5c shows that positive protein-protein interactions exist between CRMP4 and Akt or Rac1. Additionally, co-localization of CRMP4 with Rac1 was detected in a distinct perimembrane, but with Akt in the perinuclear region and cytoplasm (Figure 5d).

Rac1 enhances tissue invasion of prostate cancer cells by activating Rho GTPases and promoting activation of MMPs [32, 33], and Akt phosphorylates Rac1 at Ser71 to inhibit its GTPase activity [34, 35]. As expected, the active GTP-bound Rac1 was blocked and MMP-9 activity was markedly suppressed by CRMP4-TAL-Tet1c in PC3 cells (Figures 5e–5f). These findings were supported by immunohistochemistry data showing a differential expression pattern between CRMP4 and MMP-9 (Figure 5g).

Taken together, the results suggest that CRMP4 down-regulates MMP-9 expression and inhibits Rac1 GTPase by enhancing phosphorylation of Rac1 and Akt through direct interaction with Rac1 and Akt, eventually leading to suppression of prostate cancer invasion and metastasis (Figure 5h). Since CRMP4 lacks a kinase domain, the CRMP4-mediated phosphorylation of Rac1 and Akt must involve more signaling molecules. This demands further investigation.

### Prognostic and diagnostic significance of CRMP4 promoter methylation

**Figure 6:**
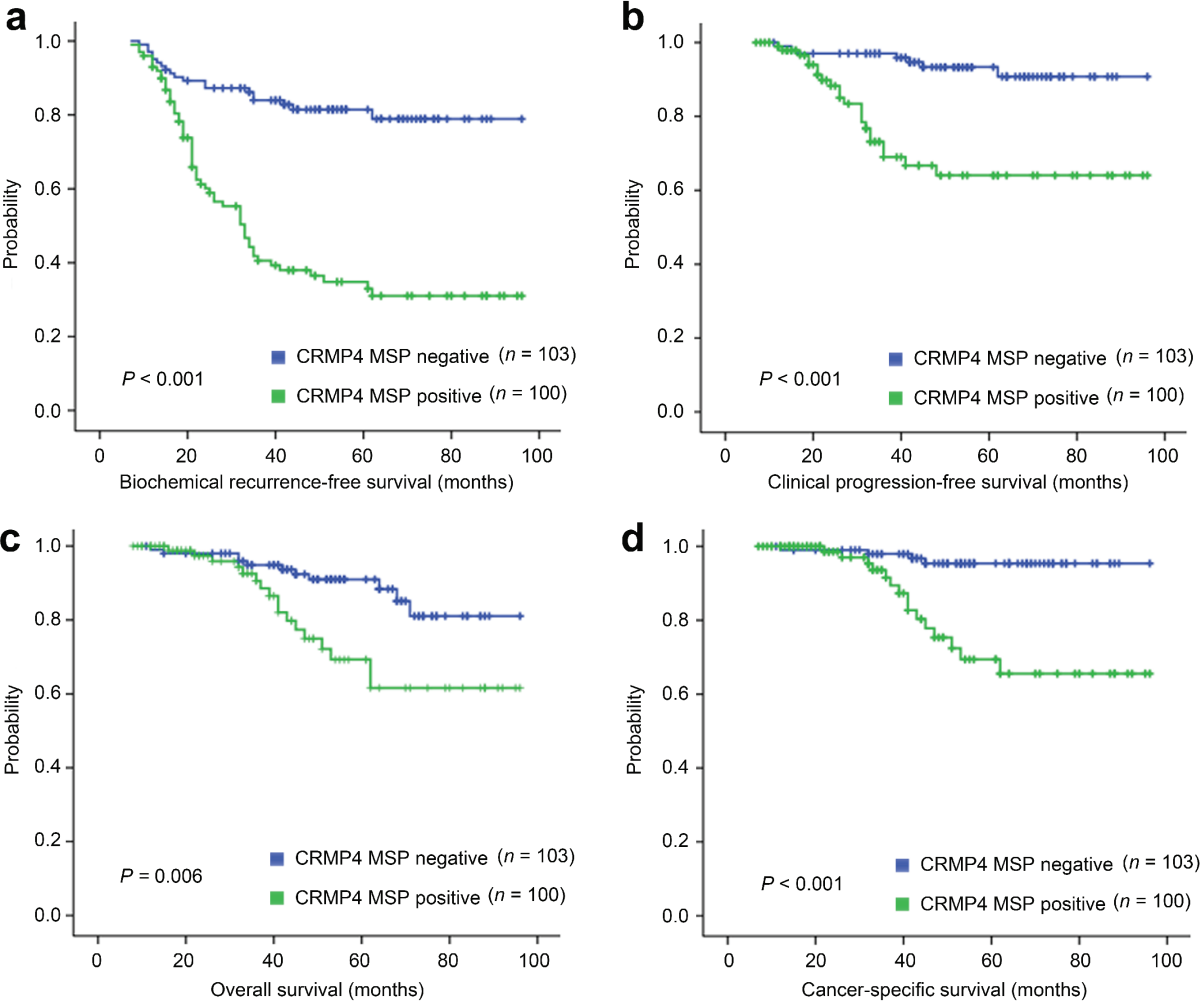
Differential survival of prostate cancer patients with positive and negative CRMP4 CpG methylation (**a**–**d**) Kaplan–Meier graphs representing the probability of cumulative **(a)** biochemical recurrence-free survival, **(b)** clinical progression-free (free of a biopsy-proven local recurrence or imaging-identified systemic metastasis lesions) survival, **(c)** overall survival and **(d)** prostate cancer-specific survival in prostate cancer patients stratified according to positive and negative CRMP4 CpG methylation status in their primary tumors. The log-rank test *P* value reflects the significance of the correlation between CRMP4 CpG methylation and survival outcome. MSP: methylation-specific PCR.

To assess whether the CRMP4 promoter methylation status would be useful for prognosis or diagnosis of metastasis in prostate cancer patients, prostate cancer specimens obtained via radical prostatectomy from 203 patients (Supplementary Table S2) were evaluated for their CRMP4 promoter methylation status using a methylation-specific PCR (MSP) method. While 103 patients were CRMP4 promoter methylation negative, 100 were CRMP4 promoter methylation-positive. Survival analysis showed that the latter had poorer biochemical recurrence-free survival (31.0% vs. 78.9%, Figure 6a), clinical progression-free survival (62.5% vs. 90.8%, Figure 6b), overall survival (61.6% vs. 81.3%, Figure 6c), and prostate cancer-specific survival (66.2% vs. 95.4%, Figure 6d). Similar to the Gleason score, pathological stage, surgical margin status and lymph node status, the multivariate Cox regression modeling results support CRMP4 CpG methylation status to be an independent prognostic factor for subsequent biochemical recurrence after radical prostatectomy (Supplementary Table S3). Among these independent prognostic factors, the CRMP4 promoter methylation status had the highest hazard ratio of 6.35 (95% CI: 4.64–8.95).

Of the 100 CRMP4 CpG methylation positive patients, 64 cases (64.0%) were clinically confirmed as metastatic cancer patients, while only one case (0.97%) out of 103 CRMP4 CpG methylation negative patients was clinically diagnosed as a metastatic cancer patient. This strongly suggests that the CRMP4 CpG methylation status can be also used as an independent marker for early diagnosis of prostate cancer metastasis.

## DISCUSSION

Among many genes involved in the development of metastasis, MSGs are responsible for negative regulation of metastasis by interfering with cancer cell dissemination, tissue invasion, survival, and growth [10, 11, 36–39]. The underlying molecular mechanisms employed by MSGs remain obscure. CRMP4 appears to act as a master negative regulator via which phosphorylation of Rac1 and Akt, and down-regulation of MMP-9 and VEGF [15], converge to suppress the development of metastasis. The metastasis-suppressing role of this novel signaling pathway identified for CRMP4 in this study (Figure 5h) is in agreement with reports implicating that Akt inhibits Rac1 activity by direct phosphorylation of Rac1 [34, 35]. It is known that Rac1 enhances prostate cancer invasion through activation of Rho GTPases and MMPs [30, 32, 33]. It appears that CRMP4-mediated suppression of metastasis involves multiple signaling pathways. The phospho-antibody microarray results further elaborate this notion since CRMP4 might also regulate PPAR, WNT and NF-κB signaling pathways, and so on (Supplementary Figure S7a and Supplementary Table S1). The present finding of CRMP4 acting as a master negative regulator is of particular importance for drug development, since targeting of such a single molecule would be sufficient to exert therapeutic efficacy, as it would simultaneously block multiple signaling events. This proof-of-concept study demonstrating that prostate cancer metastasis can be switched on and off via manipulation of CRMP4 expression further supports this notion.

Consistent with the range that has been previously reported for chimeric DNA methyltransferases [22], the CpG modifications induced by the dTALEs in this study cover a total of 600 bp sequences. This is the first report that has ever utilized a chimeric DNA demethylase for epigenomic modification. However, such modifications may not reach the first exon of CRMP4. The difference in the degree of suppression between specifically and non-specifically methylated CRMP4 promoter reporters (Figure 2b) suggests that unidentified transcription factors sensitive to CpG methylation of the core promoter and the first exon may play an important role in the initiation of CRMP4 transcription. It is also possible that CpG methylation may alter the secondary DNA structure of the reporters to block binding of potential transcription factors and RNA Polymerase II complex.

Trimethylation at H3K79 that activate gene expression has been reported before [40]. In the present study, increased trimethylation at H3K27 in Region B was also accompanied by CRMP4-TAL-Tet1c-induced activation of CRMP4 expression (Figure 4b, left panel). Given the fact that the epigenetic code, if it exists, remains completely elusive, this novel finding needs further investigation.

Both CpG and histone modifications are primary epigenetic marks. However, which modification precedes the other in transcription regulation and chromatin remodeling remains unclear [41, 42]. Current evidence suggests that CpG methyl-binding proteins (MBD1, MBD2, MeCP1/2) can recruit the histone-modifying enzymes including HDAC, histone methyltransferases and demethylases to the loci [43–46]. CpG methylation could affect binding of certain transcription factors. The latter in turn can induce histone modification by interacting with other proteins [47]. Additionally, DNA methyltransferases and demethylases form complexes that can also potentially induce histone modification [48]. Our observation that artificial dTALE-induced, yet active, locus-specific CpG modifications instigated only local histone modifications provides the first direct evidence demonstrating that CpG modifications can guide histone modifications (Figures 3a–3b). In doing so, both types of modification may work together to help transform provisional epigenomic modifications, such as temporarily induced CpG modifications, into permanent and heritable epigenetic modifications through chromatin remodeling. As a result, artificial CpG modifications can be largely preserved, altered CRMP4 expressions maintained, and continued ectopic expression of dTALEs no longer needed for maintaining CRMP4 expression.

Although a number of biomarkers for disease prognosis and diagnosis have been discovered, their mechanisms of action remain largely unknown. The fact that dTALE-mediated, locus-specific epigenomic modifications can turn on and off prostate cancer metastasis *in vitro* and *in vivo* plainly shows how the CRMP4 CpG methylation status can be an independent biomarker for early clinical prognosis and early laboratory diagnosis of prostate cancer metastasis.

Most recently, artificial TALE nucleases have been successfully utilized for genome editing [49, 50]. Whether chimeric TALE methyltransferase and demethylase can have access to desired genome loci and induce epigenomic modifications is not clear. Chimeric dTALEs fused with DNA methyltransferase and, in particular, DNA demethylase, have not previously been reported. The present study has provided proof-of-principle *in vitro,* as well as *in vivo*, that locus-specific epigenomic modification can be used for treatment of a disease such as metastatic prostate cancer. For use of such therapeutics in the clinical setting, an appropriate way of drug delivery should first be developed. Off-target binding of genomic sequences is always a concern with regard to the interpretation of observed phenotypes. Since the TALE RVD code can be degenerate [24, 51], Blast search may not be able to identify degenerate off-target sequences. Fortunately, not all off-target activity or binding would necessarily produce off-target toxicity if the off-target sequence falls into a region of little importance in the regulation of gene expression. For this proof-of-concept study, our results satisfy the design criteria.

In summary, we have provided a proof-of-concept study showing that locus-specific epigenetic modifications can be achieved with the use of dTALEs. These dTALEs can be a new approach for manipulation of disease processes such as prostate cancer metastasis. Given the fact that many critical genes, including oncogenes and MSGs, bear CpG-rich regions and CpG islands, similar dTALEs targeting these genes can be engineered as potential therapeutics and research tools to treat corresponding diseases and also improve our understanding of the gene functions.

## METHODS

### Cell culture

The human metastatic CRPC cell line PC3, non-metastatic prostate cancer cell line 22Rv1, normal prostate epithelial cell line RWPE-1, and other cell lines, i.e. HEK293 and COS-1, were obtained from the ATCC and cultured according to the supplier's instructions.

### Plasmid construction and *in vitro* CpG methylation

Based on a CRMP4 core promoter predicted with Proscan (http://www-bimas.cit.nih.gov/molbio/proscan/) (Supplementary Figure S1a), four CRMP4 promoter regions, referred to as CRMP4 A+ (−867/+114), CRMP4 A− (−839/+114), CRMP4 B+ (−717/+114), and CRMP4 B− (−656/+114), were amplified from genomic DNA of RWPE-1 cells using specific primers (Supplementary Methods). The PCR products were cloned into the pGL4.15-Luc2P (Promega) vector to construct the reporters pGL4-CRMP4-A+, pGL4-CRMP4-A−, pGL4-CRMP4-B+, and pGL4-CRMP4-B−, respectively (Figure 1a). In addition, the CRMP4-Luc2p pGL4.27 reporter was constructed by cloning one copy of the 23 bp dTALE-targeting sequence into pGL4.27 vector (Promega) that carries only a minimal promoter. The CRMP4-pCpGL reporter was constructed by cloning the CRMP4 promoter region −896/+28 into the CpG-free pCpGL vector (a gift from Michael Rehli) [52].

All reporters, except for CRMP4-Luc2p pGL4.27, were treated with non-specific CpG methyltransferase M.SssI (http://NEB.com) according to the manufacturer's instructions and the methylation was confirmed by *Hae II* digestion (http://NEB.com).

Based on a variety of criteria such as CpG distribution, distance from core promoter and transcription start site, preferred range of epigenomic modification, TALE binding requirements, and minimal homology to human genome sequence, a 23 bp sequence (TCCATTTTCTAATGTGTATGTTC) between Region A and Region B (−835/−813) was selected for dTALE-targeting (Supplementary Figures S1a, S1b). Then, a gene encoding a TALE DNA-binding domain consisting of a truncated N-terminal with a nuclear localization signal (NLS), a middle tandem repeat domain, and a C-terminal linker was commercially synthesized by http://GenScript.com with genetic codons optimized for mammalian cell expression (Supplementary Figure S2a). The RVD codes used for nucleotides A, C, G, and T were amino acids NI, HD, NN, and NG, respectively [53, 54]. The synthetic TALE DNA-binding domain was then fused to HA-Tag, transcription activation domain vp64, the catalytic domain of human methyltransferase DNMT3A (598–908 aa) and demethylase Tet1 (1612–2136 aa) to generate CRMP4-TAL-HA, CRMP4-TAL-vp64, CRMP4-TAL-3Ac, and CRMP4-TAL-Tet1c in phCMV1 vector, respectively (Figure 2a).

All the insert fragments and genes described above were verified by DNA sequencing.

### Transfection and luciferase activity assay

Transfection of single and multiple plasmids in specified cells were carried out using Lipofectamine 2000 (Invitrogen) according to the manufacturer's instructions. For PC3, 22Rv1 and HEK293 cells, 500 ng/ml Gentamicin (G418, Life Technologies) was added for 7 or 10 days to select the transfectants. Then alteration of CpG and histone methylation was analyzed with pyrosequencing (Pyromark ID96 system, Biotage) as previous reported [55] and ChIP assay [56]. All primers were summarized in supplementary appendix.

For locus-specific CpG modifications of the CRMP4-pCpGL reporter, subsequent co-transfection of the reporters and dTALEs after G418 treatment for 10 days was performed to minimize the reporter expression prior to dTALE expression. In other words, dTALEs should have already been expressed before the expression of the CRMP4-pCpGL reporter.

About 24 hours after the transfection, the luciferase activities were assayed, using a Dual Glo luciferase kit (Promega), in specified cells that were co-transfected with dual luciferase reporters alone or together with dTALEs, following the manufacturer's instructions. All data were normalized as relative firefly luciferase light/renilla units.

### Chromatin immunoprecipitation (ChIP)

ChIP assay was performed as described previously [55] using ChIP Assay Kit (Thermo). The PC3 cells transfected with CRMP4-TAL-Tet1c and 22Rv1 cells transfected with CRMP4-TAL-3Ac were lysed and the lysates processed following standard ChIP protocol. Then, qRT-PCR, as previously reported [15], was performed with the SYBR Green master mix (Toyobo), and enrichment was calculated using the percentage-of-input method.

### RNAi

The CRMP4 siRNA sequence (s4273) 5′-GGCUUAUAAGGAUUUGUAUTT-3′ was purchased from Ambion. For the study, 30 nM of CRMP4 siRNA were transfected into PC3 cells expressing CRMP4-TAL-Tet1c using siPORT NexoFX (Applied Biosystems) according to the manufacturer's procedure.

### Immunoprecipitation (IP) and western blotting

The PC3 cells expressing CRMP4-TAL-Tet1c were harvested 48 h after transfection and utilized for IP and Co-IP with Co-IP Kit (Thermo) according to the manufacturer's protocol. Specifically, the affinity-purified antibody was incubated with Amino Link Plus Coupling Resin, and then followed by immunoblot with anti-Rac1, anti-Akt or anti-CRMP4.

Western blot analysis was performed as previously reported [15] using antibodies against CRMP4 (Abcam), phospho-Akt (Ser473), phospho-Rac1 (Ser71), total-Akt and total-Rac1 (CST). GAPDH was used as an internal control in all blotting membranes (CST).

### Pull-down assays

The *in vitro* pull-down assay was performed with a Rac1 Pull-Down Kit (Thermo) according to the manufacturer's protocol. In brief, the PC3 cells transfected with and without CRMP4-TAL-Tet1c were lysed in the presence of GTPγS (positive control) or GDP (negative control). The lysates were incubated with sepharose beads. The proteins bound to the beads were analyzed using anti-Rac1 antibody by immunoblotting.

### Confocal microscopy

The PC3 cells pre-seeded on cover slips were fixed and permeabilized. Then, anti-CRMP4, anti-Rac1, and anti-Akt antibodies were incubated with the cells for 1 h, followed by addition of FITC-conjugated secondary antibodies for 30 min. The nuclei were stained with DAPI for 10 min. Confocal imaging was carried out with Zeiss LSM410 confocal microscopy systems.

### *In vitro* migration and invasion assays

The migration and tissue invasion activity of PC3 cells expressing TAL-Tet1c and 22Rv1 cells expressing CRMP4-TAL-3Ac were assessed using transwell migration assays and matrigel invasion assays as previously described [15]. Mock transfected PC3 and 22Rv1 cells were used as controls.

### *In vivo* metastasis assay, immunohistochemistry and gelatin zymographic assay

Male BALB/c nude mice (6 weeks of age) were obtained from the Jackson Laboratory. All animal experiments were approved by the Institutional Animal Care and Use Committee at Sun Yat-Sen University and performed according to its guidelines. Orthotopic implantations were carried out as previously described [15]. Briefly, the PC3 cells expressing CRMP4-TAL-Tet1c and 22Rv1 cells expressing CRMP4-TAL-3Ac were infected with LP-RLUC-LV (GeneCopoeia) containing luciferase reporter gene. The PC3 and 22Rv1 cells expressing mock vector (empty vector and LP-RLUC-LV) were used as controls. Tumor growth and metastasis distribution were assessed every 10 days using a Caliper IVIS100 imaging system (Caliper Life Science) for bioluminescence imaging. The total flux was quantified using Living Image Software v4.3.1 (Xenogen) as previously described [57].

All mice were euthanized 60 days after tumor cell implantation due to heavy primary tumor burdens. After euthanasia, *ex vivo* bioluminescence imaging (BLI) was performed to identify the location of tumors in these animals by incubation of injected luciferin for 5 min.

Tissue samples were fixed in 10% buffered formalin overnight and then embedded in paraffin, sectioned and stained with hematoxylin & eosin. The immunohistochemistry was performed using antibodies against CRMP4 and MMP-9 (Chemicon) as described previously [15]. Gelatin zymographic assay for MMP-9 activity was performed as described previously [58].

### Population study

A total of 203 prostatectomy samples from May 2004 through May 2011 in our institute were obtained for CRMP4 gene methylation assessment using MSP as previously reported [15]. This study protocol was reviewed and approved by the Institutional Review Boards at Sun Yat-Sen University. All men had received study information and signed their informed consent. Patients without previous cancer therapy were considered to be eligible in this study. The details of the baseline clinical and pathological characteristics of this cohort of patients are summarized in Supplementary Table S2. Postoperative follow-up was performed quarterly in the first year, semiannually for the second year and annually thereafter by clinical evaluation, measurement of serum PSA levels and other investigations (e.g., DRE) as indicated (median follow-up time of 48 months; longest follow-up time, 96 months). Biochemical progression was defined as a serum PSA level ≥ 0.2 ng/ml on 2 successive measurements in 3 months after surgery. Clinical progression was defined as the development of a biopsy-proven local recurrence or imaging-identified systemic metastatic lesions. Death resulting from prostate cancer or cancer-related events was defined as a cancer-specific mortality.

### Statistical analysis

One-way ANOVA was used to analyze the difference among CRMP4 promoter-driven luciferase reporters designated. Independent continuous samples were compared using the Student's *t*-test; otherwise, the Sum rank tests were employed for non-continuous variables. The χ2 test was used to evaluate the association of CRMP4 methylation results with clinical and pathologic characteristics. The survival analyses were calculated by the Kaplan–Meier method with log rank test for significance. A double-sided *p* value < 0.05 was considered statistically significant. All statistical analyses were performed using SPSS 16.0 software package (SPSS, Chicago, Illinois, USA).

## SUPPLEMENTARY METHODS, FIGURES AND TABLES


