# Supplementary material for: Manipulation of prostate cancer metastasis by locus-specific modification of the CRMP4 promoter region using chimeric TALE DNA methyltransferase and demethylase
Source: Oncotarget. 2015 Apr 9;6(12):10030–44. doi: 10.18632/oncotarget.3192 (PMC4496338; doi:10.18632/oncotarget.3192)
Supplement: Supplementary file 1 [file oncotarget-06-10030-s001.pdf]

SUPPLEMENTARY METHODS

Primers for CRMP4 promoter segments (F: forward primer)

CRMP4 A+ **F**: CCCTTGCAGCCTCTGAGAGCG AGTCACGG
CRMP4 A− **F**: GCTTTCCATTTTCTAATGTGTA TGTTCCGGG
CRMP4 B+ **F**: AGCAGAGGCGGCGCCCAGCCC
CRMP4 B− **F**: GAAGCCGGGAACCGTCTCCA TTCTG
A common reverse primer: CCGAGCCGACCCCGC CCGCAG

Primers for qRT-PCR (F: forward primer; R: reverse primer)

CRMP4 **F**: CGTCCACTTCTGCACTCTCT
CRMP4 **R**: AAAGGTTCTGGTGGAATGGT
Akt1 **F**: CTATGGCGCTGAGATTGTG
Akt1 **R**: CTTAATGTGCCCGTCCTTGT
Akt2 **F**: TGAAAACCTTCTGTGGGACC
Akt2 **R**: TGGTCCTGGTTGTAGAAGGG
Akt3 **F**: GGCGAGCTGTTTTTCCATTTG
Akt3 **R**: GGCCATCTTTGTCCAGCATTG
Rac1 **F**: CCCCCCATTCTTGTTCAGATT
Rac1 **R**: TGCTTTACGCATCTGAGAACTACAT
18s rRNA **F**: CCTGGATACCGCAGCTAGGA
18s rRNA **R**: GCGGCGCAATACGAATGCCCC

Primers for ChIP (F: forward primer; R: reverse primer)

CRMP4 ChIP **F**1: ACGCGGGTGGAGGAGGGTGTG
CRMP4 ChIP **R**1: CCCGAGATCAGGTGGAGTGA
CRMP4 ChIP **F**2: GAATGGTTGAGTCCACTGCTA
CRMP4 ChIP **R**2: CCCGGAACATACACATTAGA
CRMP4 ChIP **F**3: GCTGCGCCGCTGTTTACC
CRMP4 ChIP **R**3: TACCTTCCCCGCCCTAGTTTCA
CRMP4 ChIP **F**4: CAGTTCTACTCCAAATCCATG
CRMP4 ChIP **R**4: CCCGGAACATACACATTAGAA
CRMP4 ChIP **F**5: CTAATGTGTATGTTCCGGGTC
CRMP4 ChIP **R**5: GTTTCAGAATGGAGACGGTTC
GAPDH ChIP **F**: CTCCTACCAGAAGAATGGATCC
GAPDH ChIP **R**: CTTTCATTCCATCCAGCCT GGG
RASSF1A ChIP **F**: CCAAGTGTGTTGCTTCAGCA AAC
RASSF1A ChIP **R**: CTTGCCCTTCCTTCCCTCC TTC
p16 ChIP **F**: CTCCTGAAAATCAAGGGTTGAGG
p16 ChIP **R**: CTTCCTAACTGCCAAATTGAATCG

Primers for pyrosequencing

The sequence of 300 bp in both directions from the TALE-targeting sequence (−835/−813) was respectively amplified by PCR using the specific primers PCR-1 and PCR-2 for further pyrosequencing toward 5′ of Region A and 3′ of Region B using sequencing primers
PCR-1 forward: ATAGGGAATGGTTGAGTTTAT TGT
PCR-1 reverse: Biotin-ACCCCCTCTCCTCTACCATA
Sequencing primer 1: GTTTTTTGTAGTTTTTGAGA (−873/−853)
Sequencing primer 2: AGTTTTTTTTTAGAATAAAG (−704/−685)
Sequencing primer 3: TTTAGGAGTTTGAAGGG (−766/−750)
Sequencing primer 4: GTTTAGGGTTTGGGGA (−819/−804)
Sequencing primer 5: GGGAATGGTTGAGTTTA (−899/−883)
PCR-2 forward: AGGGTTTGGGGATTTTAGTAGGT
PCR-2 reverse: Biotin-TCCCCAAAATAAAAACA TCAACT
Sequencing primer 1: TAGAGTTATGGTAGAGGA GAG (−664/−644)
Sequencing primer 2: GGGGAAGGTAGGAGAT (−594/−579)

Primers for detecting hypermethylation CpG islands in regions A and B

A forward: AGGGAATGGTTGAGTTTATTGTTA
A reverse: Biotin-ACACCCCCTCTCCTCTACCATA
Sequencing primer A: GTTTTTTGTAGTTTTTGAGA (−873/−853)
B forward: ATATAGGGAATGGTTGAGTTTATTGT
B reverse: Biotin-ACACCCCCTCTCCTCTACCATA
Sequencing primer B: AGTTTTTTTTTAGAATAAAG (−704/−685)

Primers for detecting alteration of methylation on p16, TWIST1 and RASSF1A promoter. (F: forward primer; R: reverse primer)

p16 **F**: GGAGGAAGAAAGAGGAGGGGT
p16 **R**: Biotin-CAACCAATCAACCRAAAACTC
TWIST1 **F**: GGGAGAGATGAGATATTATTTATT GTGT
TWIST1 **R**: Biotin-CTCCTCCCAAACCATTCAA
RASSF1A **F**: TAGTGGGTAGGTTAAGTGTGTTGT
RASSF1A **R**: Biotin-TACCCTTCCTTCCCTCCTTC

Phospho explorer antibody microarray

The phospho-antibody array was performed according to the manufacturer's protocol using Phospho Explorer Antibody Microarray (PEX100, Full Moon Biosystems). This antibody array contained 1318 well-characterized phospho-specific antibodies and also antibodies for phospho-specific antibodies and their non-phospho pairs to determine the relative level of phosphorylation. Each antibody is printed in duplicates. Protein was extracted from PC3 cells transfected with and without CRMP4-TAL-Tet1c, respectively. The protein samples were used as subsequent biotin labeling and chip hybridization. Chip images were scanned using Axon GenePix 4000B, and the original data were acquired by GenePix software Pro 6. For data analysis, background signals were removed from all measurements. A ratio was calculated to measure the extent of protein phosphorylation. Results from quadruplicate samples were averaged.

SUPPLEMENTARY FIGURES AND TABLES

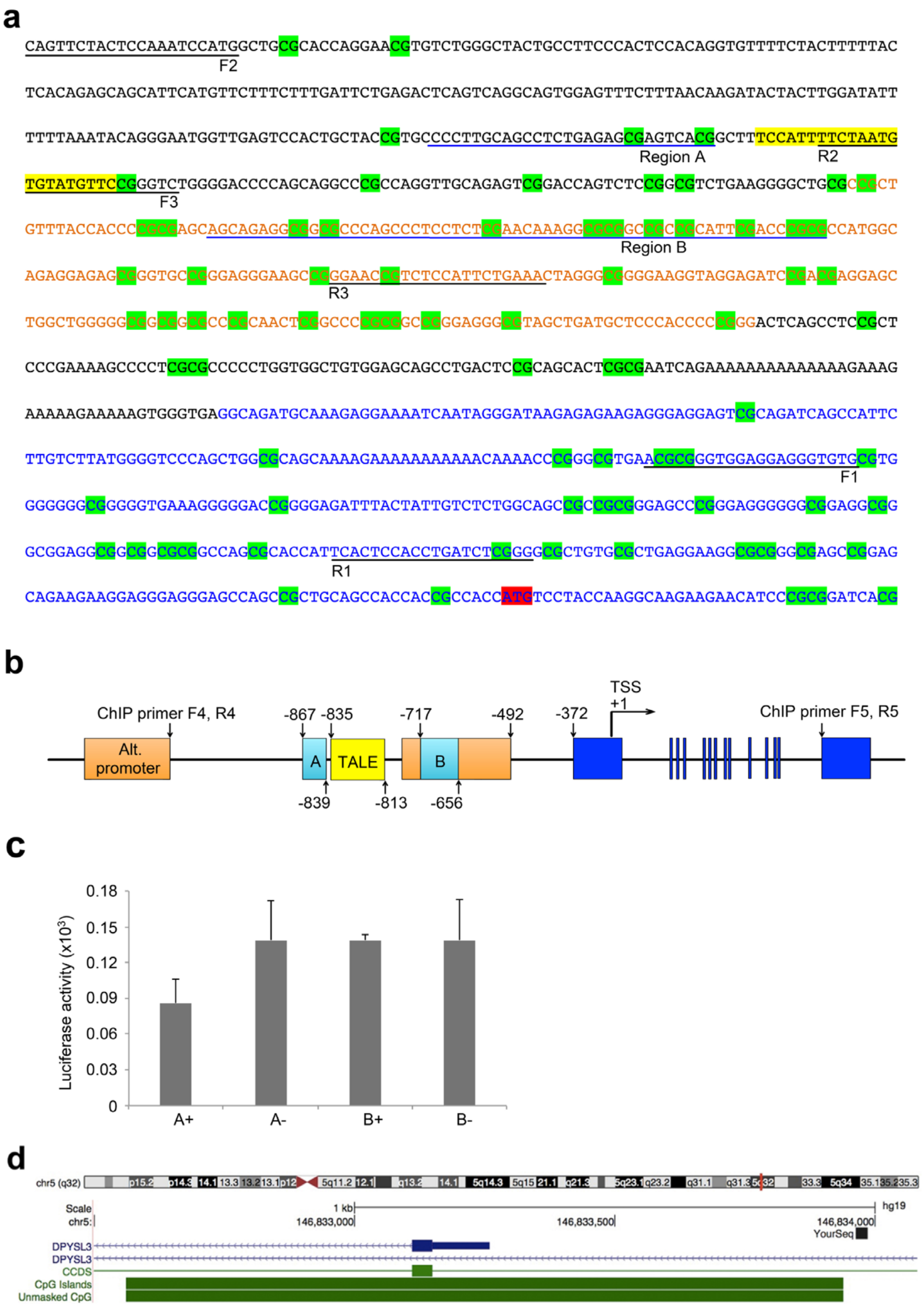

Supplementary Figure S1: Human CRMP4 gene in chromosome 5. (a) Promoter sequence of CRMP4 variant B (NM_001387). The core promoter sequence in orange was predicted with a bioinformatic software Proscan (http://www-bimas.cit.nih.gov/molbio/proscan/). Primers used for ChIP assay are underlined in black and labeled underneath. Regions A and Region B are underlined in blue and labeled underneath. All CpG sites are highlighted in green. The initial exon is in blue. The selected TALE targeting sequence is highlighted in yellow. The start codon is highlighted in red. F1-5: forward primer 1-5; R1-5: reverse primer 1-5. **(b)** Schematic of CRMP4 gene structure. CRMP4 alternative promoter for variant A (NM_001197294), the terminal exon, and their approximate ChIP detection sites are shown. The labeled sequence numbers are not on scale. The boxes shaded in orange: promoters, cyan: Regions A and B as labeled, yellow: the 23 nt TALE targeting sequence, blue: exons. **(c)** Luciferase activities of the four CRMP4 promoter reporters that were pre-treated with M.SssI. **(d)** Query of a 23 bp sequence selected as dTALE targeting modified against the human genome in Blast search. The *P* values in **c** were determined with the Student's *t*-test. The error bars in **c** are s.e.m.

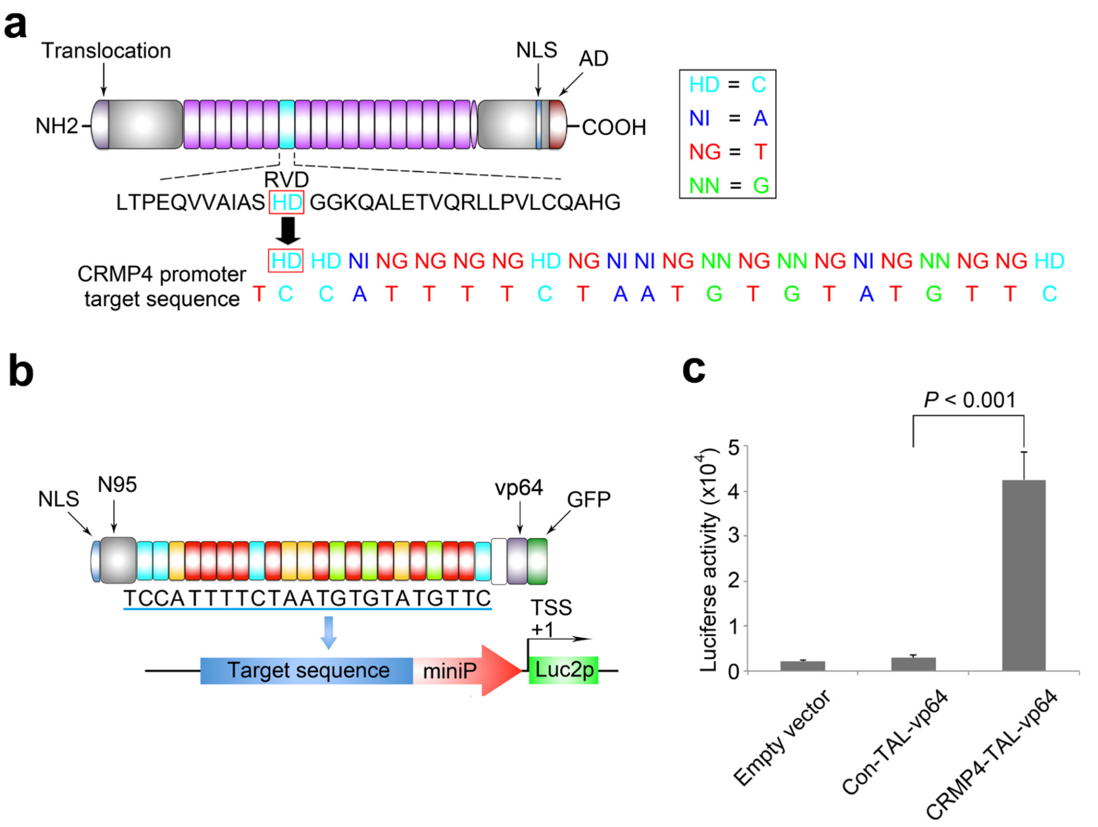

Supplementary Figure S2: Recognition of dTALEs to its targeting sequence. **(a)** Schematic of a wild-type TALE targeting the selected 23 nt CRMP4 promoter sequence. The N-terminal domain, middle tandem repeat domain, C-terminal linker, and other functional domains and motifs are shown. The modules recognizing different nucleotides are shown with corresponding colors (cyan for C, blue for A, red for T, and green for G). NLS: Nuclear Localization Signal; RVD: the Repeat Variable Di-residues; AD: transcription Activation Domain; a middle tandem repeat domain, and a C-terminal linker. **(b)** Composition of the CRMP4-Luc2p pGL4.27 reporter (lower panel) and CRMP4-TAL-vp64 (upper panel). **(c)** Luciferase activity induced by CRMP4-TAL-vp64 through targeted specific activation of the minimal promoter in the reporter. The *P* value in **c** is obtained using the Student's *t*-test. The error bars in **c** are s.e.m.

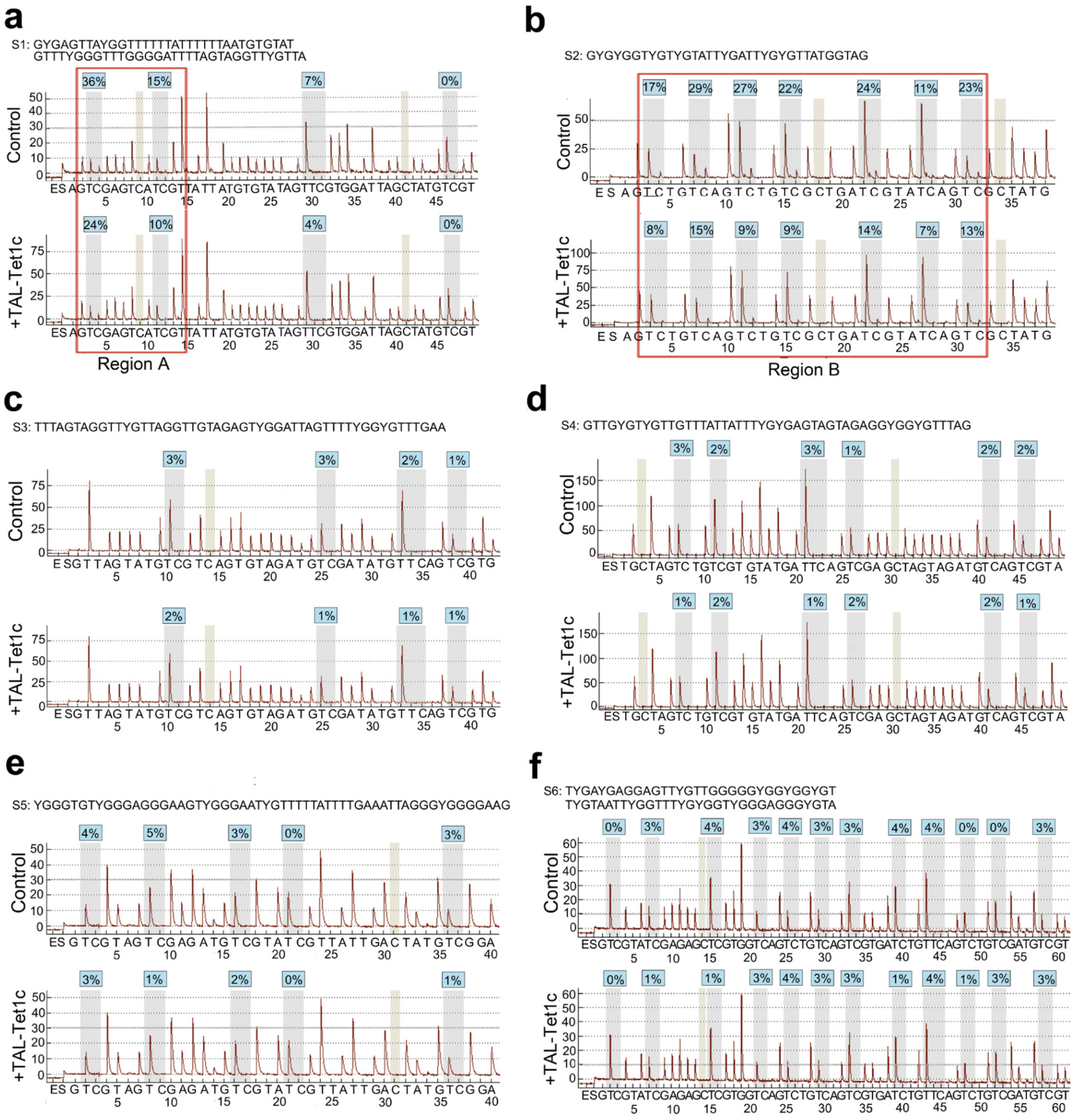

Supplementary Figure S3: The potential area in the CRMP4 promoter likely affected by CRMP4-TAL-Tet1c. Bisulfite treatment-coupled pyrosequencing analysis of Region A **(a)** and Region B **(b)** of genomic DNA isolated from the PC3 cells with and without transfection of CRMP4-TAL-Tet1c ($P = 0.003$, $P < 0.001$, respectively). **(c–f)** Bisulfite treatment-coupled pyrosequencing analysis of the CRMP4 promoter region towards the 5′ and 3′ of the CRMP4-TAL-Tet1c targeting sequence. These sequences cover about 300 bp in both directions. The CpG demethylation was detected in various degrees following transfection of CRMP4-TAL-Tet1c. The P values in **a, b** are obtained using the Student's t-test.

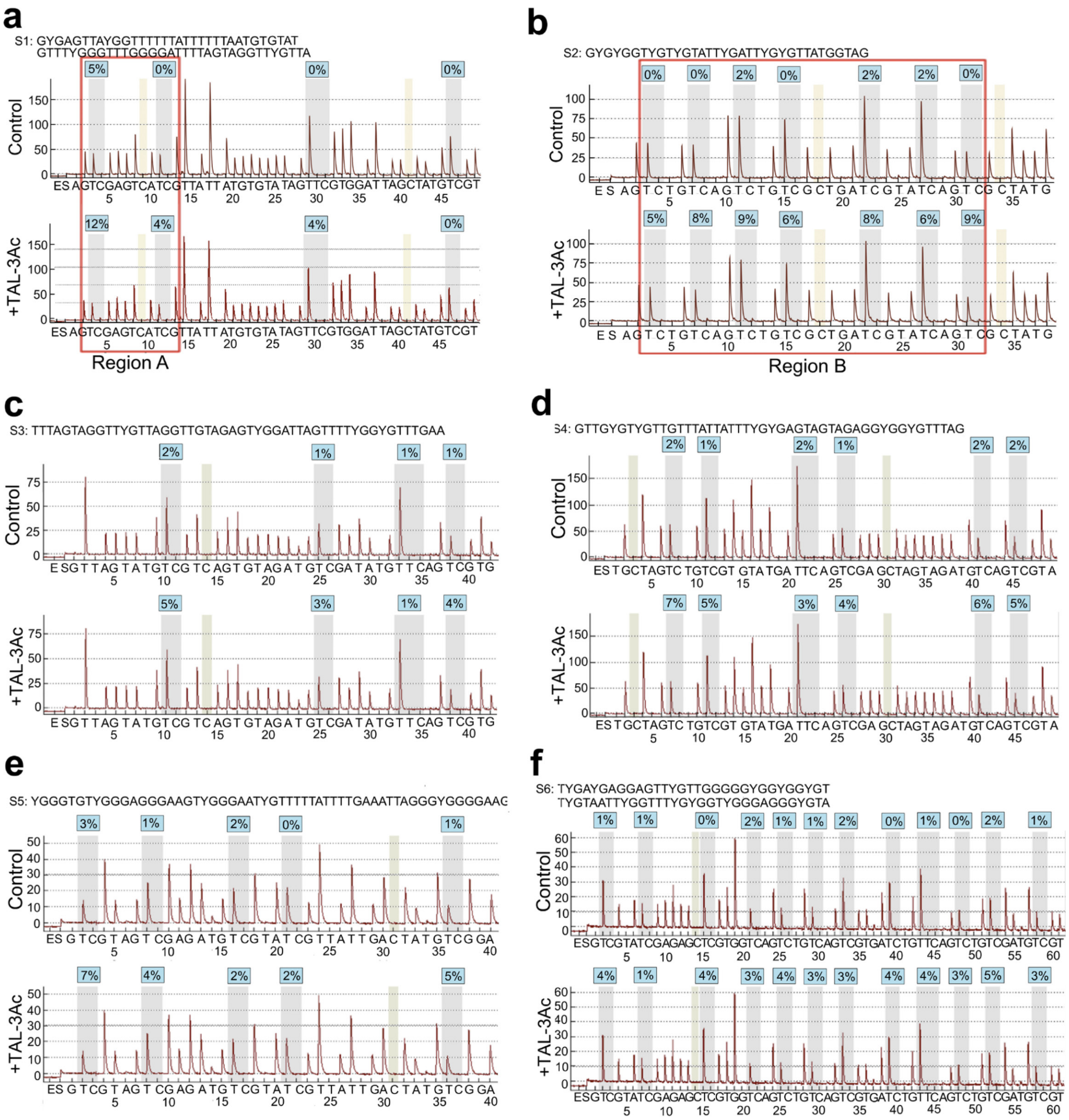

Supplementary Figure S4: The potential area in the CRMP4 promoter likely affected by CRMP4-TAL-3Ac. Bisulfite treatment-coupled pyrosequencing analysis of Region A **(a)** and Region B **(b)** of genomic DNA isolated from the 22Rv1 cells with and without transfection of CRMP4-TAL-3Ac ($P = 0.01$, $P < 0.01$, respectively). **(c–f)** Bisulfite treatment-coupled pyrosequencing analysis of CRMP4 promoter region towards the 5’ and 3’ of the CRMP4-TAL-3Ac targeting sequence. These sequences cover about 300 bp in both directions. The CpG methylation was detected in various degrees following transfection of CRMP4-TAL-3Ac. The *P* values in **a**, **b** are obtained using the Student’s *t*-test.

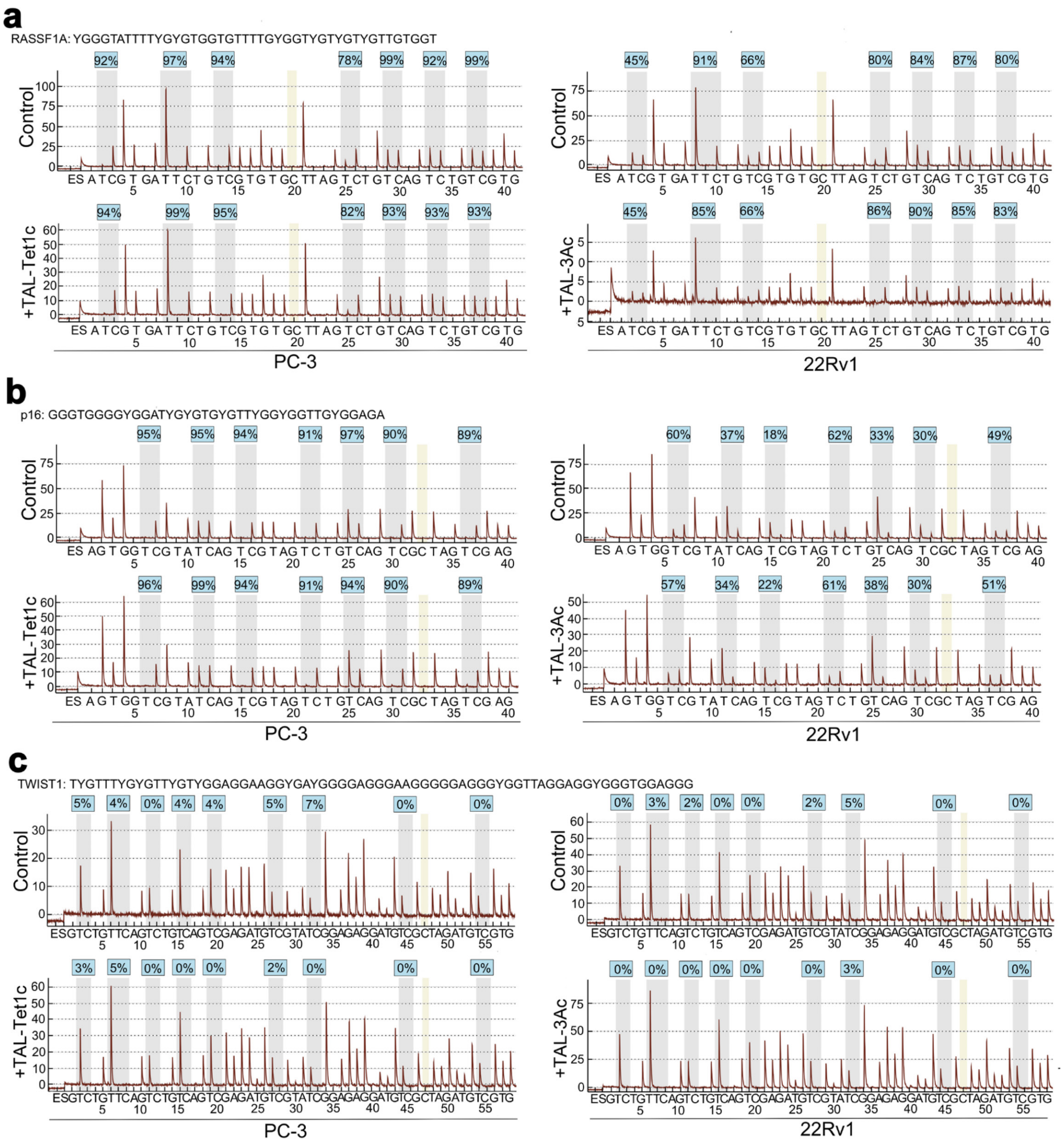

Supplementary Figure S5: Off-target CpG modifications of the control gene promoters analyzed using bisulfite treatment-coupled pyrosequencing. CpG modifications of **(a)** *RASSF1A* (NM_007182), **(b)** *p16*^{INK4A} (NM_000077), and **(c)** *TWIST1* (NM_000474) promoters in the PC3 cells (right column) and 22Rv1 cells (left column) transfected with CRMP4-TAL-Tet1c and CRMP4-TAL-3Ac, respectively. Their corresponding controls are presented. The *P* values in **a–c** obtained using the Student's *t*-test are much greater than 0.05 and thus not significant.

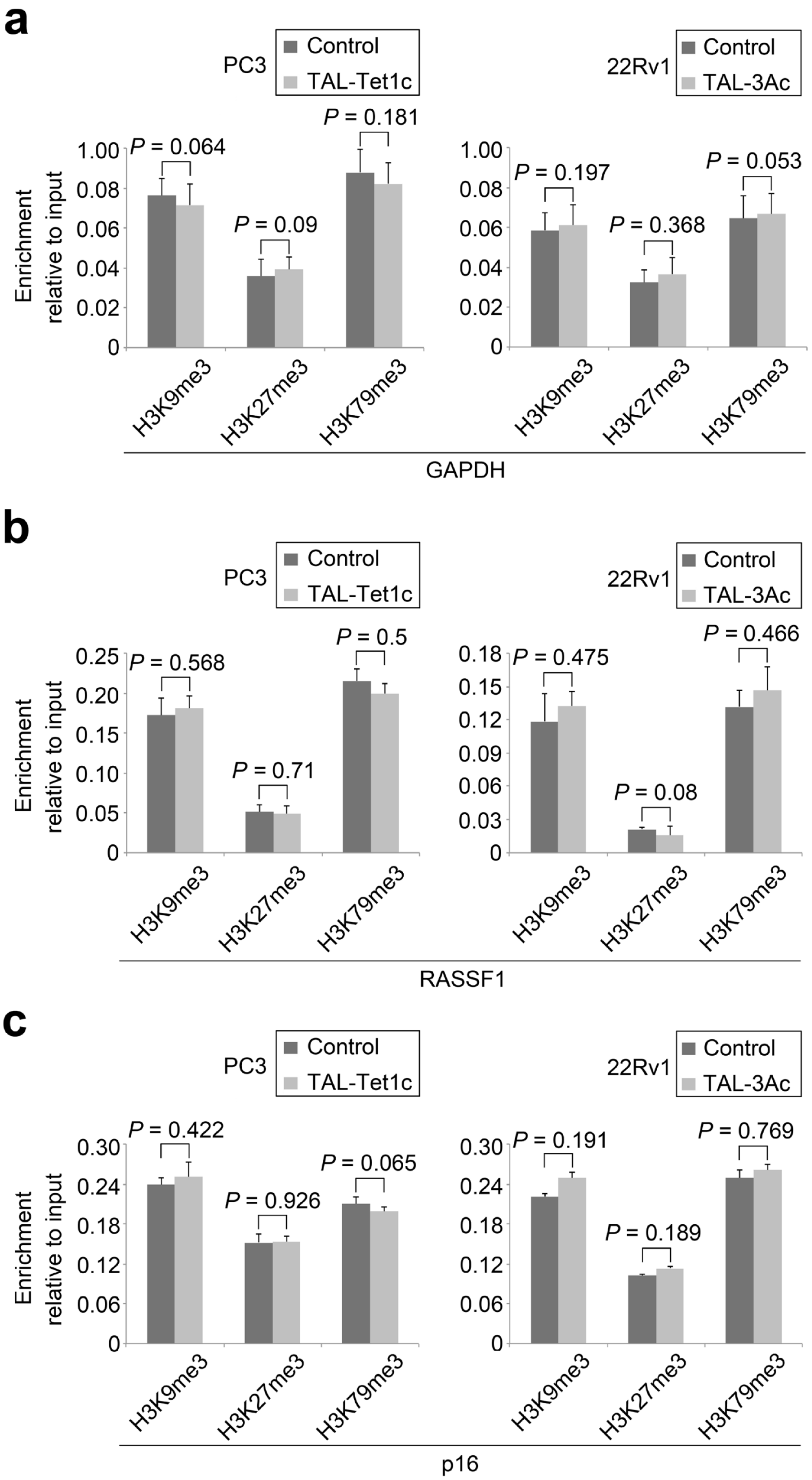

Supplemental Figure S6: Off-target histone modifications of the control gene promoters analyzed using ChIP assay. Ectopic expression of CRMP4-TAL-Tet1c in PC3 cells and CRMP4-TAL-3Ac in 22Rv1 cells induced the histone modifications at H3K9me3, H3K27me3 and H3K79me3 in **(a)** control gene *GAPDH* (NM_002046), **(b)** control gene *RASSF1A* (NM_007182), and **(c)** control gene *p16*INK4a (NM_000077). Empty phCMV1 vector was transfected in PC3 and 22Rv1 cells as controls. Different from CRMP4 that is located in chromosome 5, the three control genes are located, respectively, in chromosome 12, 3, and 9. The *P* values in **a–c** obtained using Student's *t*-test are much greater than 0.05 and thus not significant.

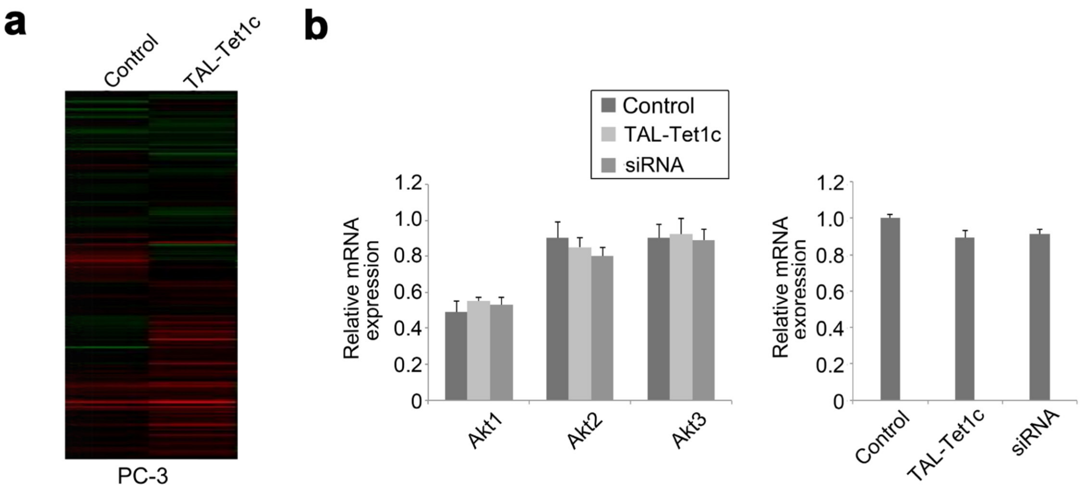

Supplementary Figure S7: Phosphorylation status and specific gene expression induced by CRMP4-TAL-Tet1c. (a) A heatmap reflecting protein phosphorylation alterations between the PC3 cells with and without transfection of CRMP4-TAL-Tet1c as detected by Phosphorylation Explorer Antibody Microarray. Akt (**b,** left panel) and Rac1 (**b,** right panel) mRNA levels in the PC3 cells with and without CRMP4-TAL-Tet1c expression were detected with no alterations using qRT-PCR analysis. The *P* values in **b** obtained using the Student's *t*-test are much greater than 0.05 and not significant.

Supplementary Table S1: Alteration of protein phosphorylation by overexpressing CRMP4

Phosphorylation Sites	Ratio	* Signaling pathways
4E-BP1 (Phospho-Ser65)	1.28	AMPK, PMT, PIG
4E-BP1 (Phospho-Ser65)	1.28	AMPK
4E-BP1 (Phospho-Ser65)	1.28	PMT
4E-BP1 (Phospho-Thr36)	1.90	AKT, ChT, MAPK, AMPK, PMT, PIG
4E-BP1 (Phospho-Thr36)	1.90	AMPK
4E-BP1 (Phospho-Thr36)	1.90	PMT
4E-BP1 (Phospho-Thr36)	1.90	PIG
Abl1 (Phospho-Tyr204)	3.95	CCC, PTG, PST, PER
ACC1 (Phospho-Ser79)	2.27	AMPK
ACC1 (Phospho-Ser80)	1.16	AMPK
ACTIN Pan (a/b/g) (Phospho-Tyr55/53)	1.12	CYT, PST
AKT (Phospho-Ser473)	1.90	AKT, PKB, MAPK, CYT, PAB, PAK, PER, PIG, PIR, PMT, PNK, PTC, PTG
AKT1 (Phospho-Ser124)	1.32	PKB, AMPK, CCC, PTG, PNK, PMT, PJS, PIR, PIG, PER
AKT1 (Phospho-Ser246)	1.25	PKB, AMPK, CCC, PTG, PNK, PMT, PJS, PIR, PIG, PER
AKT1 (Phospho-Tyr474)	1.37	PKB, AMPK, APO, CCC, CREB, PDGF, PTG, PST, PNK, PMT, PJS, PIR, PIG, PER
AKT1S1 (Phospho-Thr246)	1.53	PKB, AMPK, PNK, PMT, PJS, PIR, PIG

(*Continued*)

Phosphorylation Sites	Ratio	* Signaling pathways
AMPK1 (Phospho-Thr174)	1.30	AMPK, PMT, PIG, MET
Androgen Receptor (Phospho-Ser213)	1.21	NMR
Androgen Receptor (Phospho-Ser650)	1.16	NMR
A-RAF (Phospho-Tyr301/302)	1.10	APO, PST, PEK
Arrestin-1 (Phospho-Ser412)	1.11	PDG, PEK
ASK1 (Phospho-Ser966)	2.78	AKT, MAPK
ASK1 (Phospho-Ser966)	2.78	APO
ATF2 (Phospho-Ser112/94)	1.25	PGF
ATF4 (Phospho-Ser245)	1.43	ChT, MAPK
ATP-Citrate Lyase (Phospho-Ser454)	1.27	PIG
ATRIP (Phospho-Ser68/72)	1.51	APO, CCC, PFT
BCL-XL (Phospho-Ser62)	1.11	AKT, APO, PJS
BCL-XL (Phospho-Thr47)	1.42	APO, PJS
BCR (Phospho-Tyr177)	1.15	PST
B-RAF (Phospho-Ser446)	2.78	APO, PDG, PEK
B-RAF (Phospho-Thr598)	1.25	APO, PDG, PEK
BRCA1 (Phospho-Ser1423)	1.60	CCC, ChT, PFT
BRCA1 (Phospho-Ser1457)	1.25	CCC, PFT
BRCA1 (Phospho-Ser1524)	1.54	CCC, ChT, PFT
BTK (Phospho-Tyr222)	1.20	PST, PNK
Calmodulin (Phospho-Thr79/Ser81)	3.15	CYT, PTC, PIR, PER
CaMK1-a (Phospho-Thr177)	1.21	CYT, CREB, PIR, PER, PEK
CaMK2-beta/gamma/delta (Phospho-Thr287)	2.00	AMPK, CYT, CREB, PIR, PDG, PER, PEK
CASP8 (Phospho-Ser347)	1.25	APO
Caspase9 (Phospho-Ser196)	1.21	APO, PVE, PIR
Caspase9 (Phospho-Tyr153)	1.45	APO, PVE, PST, PIR
Catalase (Phospho-Tyr385)	1.57	PST
Cateninbeta (Phospho-Ser37)	1.84	EGF, PER, WNT
Caveolin-1 (Phospho-Tyr14)	1.36	CYT, EGF, PST, PIG, PER
CD3Z (Phospho-Tyr142)	1.38	PTC, PST
CD4 (Phospho-Ser433)	1.64	PTC
CDC25B (Phospho-Ser353)	1.21	CCC, PDG
CDC25C (Phospho-Ser216)	1.85	APO, CCC, CYT, PDG
CDK5 (Phospho-Tyr15)	2.00	PST
Chk1 (Phospho-Ser345)	1.12	CCC, APO, PFT
Chk2 (Phospho-Thr383)	2.31	APO, CCC, PFT

(*Continued*)

Phosphorylation Sites	Ratio	* Signaling pathways
Chk2 (Phospho-Thr387)	1.12	APO, CCC, PFT
Chk2 (Phospho-Thr68)	1.25	CCC, APO, PFT
c-Jun (Phospho-Ser73)	1.62	AKT, ChT, MAPK, PDGF, PTC
c-Jun (Phospho-Thr93)	1.52	AKT, ChT, MAPK, PDGF, PTC
CK2-b (Phospho-Ser209)	1.16	PNK
c-met (Phospho-Tyr1003)	1.74	PST
Cofilin (Phospho-Ser3)	1.22	CYT, PTG
Connexin43 (Phospho-Ser367)	1.28	CYT
Cortactin (Phospho-Tyr421)	1.42	CYT, PST
Cortactin (Phospho-Tyr466)	1.69	CYT, PST
c-Raf (Phospho-Ser43)	1.40	CYT, CREB, EGF, MET, PJS, PIR, PIG, PDG, PFT, PEK
CREB (Phospho-Ser121)	1.13	CREB, PIR, PGF, PEK
CREB (Phospho-Ser133)	1.32	APO, ChT, MAPK, CREB, PIR, PGF, PEK
CREB (Phospho-Ser142)	1.20	CREB, PIR, PGF, PEK
CyclinB1 (phospho-Ser126)	2.56	AMPK, CCC
CyclinD1 (Phospho-Thr286)	2.68	PKB, CCC, PFT
CyclinD3 (Phospho-Thr283)	1.21	CCC, PFT
CyclinE1 (Phospho-Thr395)	1.12	CCC, PFT
DAXX (Phospho-Ser668)	1.72	APO
DDX5/DEAD-boxprotein5 (Phospho-Tyr593)	1.55	PST
DNA-PK (Phospho-Thr2638)	1.36	CCC, PFT
Dok-2 (Phospho-Tyr299)	1.74	EGF, PER
DYN1 (Phospho-Ser774)	1.87	PDG, PEK
E2F1 (Phospho-Thr433)	1.31	CCC, PFT
EEF2 (Phospho-Thr56)	1.19	AMPK
eEF2K (Phospho-Ser366)	1.49	AMPK
EGFR (Phospho-Tyr1016)	1.15	EGF, PST, PJS
EGFR (Phospho-Tyr1016)	1.15	PER
EPHA2/3/4 (Phospho-Tyr588/596)	1.30	PST
FADD (Phospho-Ser194)	1.24	APO
Filamin A (Phospho-Ser2152)	1.31	CYT
FosB (Phospho-Ser27)	1.47	CREB, PDGF, PTC, PEK
FOXO1A (Phospho-Ser329)	1.76	PKB, APO, MET, CCC, PIG, PER
Gab1 (Phospho-Tyr627)	1.53	AKT, PKB, EGF, PST, PIG, PGF, PER
GAB1 (Phospho-Tyr659)	1.37	PKB, EGF, PST, PIG, PGF, PER

(*Continued*)

Phosphorylation Sites	Ratio	* Signaling pathways
GABA-RB (Phospho-Ser434)	1.20	PKB, PGF
GAP43 (Phospho-Ser41)	1.16	PDGF
GRB10/Growth factor receptor-bound protein 10 (Phospho-Tyr67)	1.32	PST, PIG
GRK2 (Phospho-Ser29)	1.90	PDG
HCK (Phospho-Tyr410)	1.16	PST
HDAC1 (Phospho-Ser421)	1.15	CCC, PNK, PFT, WNT
HDAC3 (Phospho-Ser424)	1.60	CCC, PNK, PFT, WNT
HDAC4 (Phospho-Ser632)	1.12	CCC, ChT, PNK, PFT, WNT
HDAC5 (Phospho-Ser259)	1.56	CCC, PNK, PFT, WNT
HDAC5 (Phospho-Ser498)	1.12	CCC, ChT, PNK, PFT, WNT
HDAC6 (Phospho-Ser22)	1.27	CCC, PNK, PFT, WNT
HER2 (Phospho-Tyr1221/Tyr1222)	3.05	EGF, PER
HER2 (Phospho-Tyr877)	1.84	NMR, EGF, PST, PER
HER3/ErbB3 (Phospho-Tyr1289)	1.26	PST, PER
HRS (Phospho-Tyr334)	1.32	PST
HSL (Phospho-Ser552/563)	1.69	AMPK, PIG
HSL (Phospho-Ser554)	1.26	AMPK, PIG
HSP27 (Phospho-Ser82)	2.13	APO, MAPK, APO, PVE
IkB-beta (Phospho-Thr19)	2.79	APO, PDGF, PTC, PNK
IKK-gamma (Phospho-Ser31)	1.50	APO, EGF, PIR
IKK-gamma (Phospho-Ser85)	3.57	APO, PDGF, EGF, PTC, PNK, PIG
Integrinbeta-1 (phospho-Thr788)	1.31	PDG
Interferon-gamma receptor alpha chain precursor (Phospho-Tyr457)	1.21	PST, PJS
IR (Phospho-Tyr1361)	1.15	PST, PMT, PIG
IRS-1 (Phospho-Ser312)	1.42	AKT, APO, MAPK, PKB, PIR, PIG
IRS-1 (Phospho-Ser323)	2.28	PKB, PIR, PIG
IRS-1 (Phospho-Ser639)	2.54	AKT, APO, MAPK, PKB, PIR, PIG
JAK1 (Phospho-Tyr1022)	1.16	AKT, APO, PKB, PDGF, EGF, PST, PJS, PER
JunB (Phospho-Ser79)	1.14	ChT, MAPK
LaminA/B (laminA/C) (Phospho-Ser392)	1.45	APO
LAT (Phospho-Tyr171)	1.31	PTC, PST
LAT (Phospho-Tyr191)	1.89	PTC, PST
LCK (Phospho-Ser59)	1.11	PTC, PNK
Lck (Phospho-Tyr393)	1.51	PTC, PST, PNK
LIMK1 (Phospho-Thr508)	1.20	CYT, PTG

(*Continued*)

Phosphorylation Sites	Ratio	* Signaling pathways
LKB1 (Phospho-Ser428)	1.21	AMPK, PIG
LYN (Phospho-Tyr507)	1.71	PKB, PST
MAPK APK2 (Phospho-Thr334)	2.47	PVE, PDG, PGF, PFT
MDM2 (Phospho-Ser166)	1.57	PKB, CCC, PFT, PER
MEF2A (Phospho-Thr312)	1.13	ChT, MAPK, AMPK, PIR, PER
MEF2A (Phospho-Thr319)	1.91	ChT, MAPK, AMPK, PIR, PER
MEK1 (Phospho-Ser217)	1.11	CYT, MAPK, CYT, MET, CREB, PDGF, EGF, PVE, PTC, PJS, PIG, PDG, PGF, PFT, PEK
MEK1 (Phospho-Ser298)	2.29	CYT, CREB, PDGF, MET, EGF, PVE, PTC, PJS, PIG, PDG, PGF, PFT, PEK
MEK1 (Phospho-Thr286)	1.12	CYT, CREB, PDGF, EGF, MET, PVE, PTC, PJS, PIG, PDG, PGF, PFT, PEK
Merlin (Phospho-Ser10)	2.41	CYT
Merlin (Phospho-Ser518)	1.27	CYT
Met (Phospho-Tyr1349)	1.73	AKT, MAPK, PST
MKK3 (Phospho-Ser189	1.13	EGF, PVE
MKK6 (Phospho-Ser207)	1.35	EGF, PVE, PTG, PTC
MKP-1 (Phospho-Ser359)	1.18	PDG
MKP-1/2 (Phospho-Ser296)	1.14	PDG
Mnk1 (Phospho-Thr385)	1.19	PMT, PEK
MSK1 (Phospho-Ser360)	1.30	PNK, PDG, PEK
mTOR (Phospho-Ser2448)	1.16	PKB, AMPK, MET, PTG, PMT, PJS, PIR, PIG, PER
mTOR (Phospho-Ser2481)	1.36	PKB, AMPK, MET, PTG, PMT, PJS, PIR, PIG, PER
Myc (Phospho-Ser373)	1.25	CCC, ChT, MAPK, PTG, PJS, PEK
Myc (Phospho-Thr58)	1.54	CCC, ChT, MAPK, PTG, PJS, PEK
NFkB-p100/p52 (Phospho-Ser865)	2.09	APO, PDGF, PNK
NFkB-p100/p52 (Phospho-Ser869)	1.81	APO, PNK
NFkB-p105 (Phospho-Ser927)	1.22	APO, PDGF, PTC, PNK
NFkB-p105/p50 (Phospho-Ser337)	1.18	APO, PDGF, PNK
NFkB-p105/p50 (Phospho-Ser907)	1.34	APO, PNK
NFkB-p105/p50 (Phospho-Ser932)	1.90	APO, PDGF, PNK
NFkB-p65 (Phospho-Ser276)	1.25	APO, PNK
NFkB-p65 (Phospho-Ser529)	1.43	APO, PNK
NFkB-p65 (Phospho-Thr254)	1.12	APO, PDGF, PNK
NMDAR2B (Phospho-Tyr1472)	2.07	PST, PER
p130Cas (Phospho-Tyr165)	1.18	CYT, PST, PER

(*Continued*)

Phosphorylation Sites	Ratio	* Signaling pathways
p53 (Phospho-Ser15)	1.40	AKT, APO, ChT, MAPK, PKB, AMPK, CCC, PFT, PER
p53 (Phospho-Ser20)	1.53	PKB, AMPK, CCC, PFT
p53 (Phospho-Ser315)	1.92	AKT, APO, ChT, MAPK, PKB, AMPK, CCC, PFT
p53 (Phospho-Ser33)	1.22	AKT, APO, ChT, MAPK, PKB, AMPK, CCC, PFT
p53 (Phospho-Ser378)	1.14	PKB, AMPK, CCC, PFT
p53 (Phospho-Ser392)	1.18	PKB, AMPK, APO, CCC, PFT, PER
p53 (Phospho-Ser9)	1.69	AKT, APO, ChT, MAPK, PKB, AMPK, CCC, PFT
P70S6K (Phospho-Ser418)	1.29	PKB, AMPK, APO, PMT, PIR, PIG
P70S6K (Phospho-Thr229)	1.24	PKB/AMPK/APO/PMT/PIR/PIG/PER
P70S6k (Phospho-Thr421)	1.81	PKB, AMPK, APO, PMT, PIR, PIG
P70S6k-beta (Phospho-Ser423)	1.29	PKB, APO, PMT, PIR, PER
P90RSK (Phospho-Thr573)	1.12	APO, CCC, PMT, PDG
P95/NBS1 (Phospho-Ser343)	1.15	CCC
PAK1 (Phospho-Thr212)	1.24	PDGF, EGF, PTG, PER, PEK
PAK1/2 (Phospho-Ser199)	1.29	PDGF, EGF, PTG, PER, PEK
PAK1/2/3 (Phospho-Thr423/402/421)	2.03	PDGF, EGF, PTG, PEK
PAK2 (Phospho-Ser192)	1.20	PDGF, PTG, PEK
PDK1 (Phospho-Ser241)	1.65	AKT, CYT, PKB, EGF, PMT, PIR, MET, PIG
PECAM-1 (Phospho-Tyr713)	1.58	PST
PKACAT (Phospho-Thr197)	1.18	AMPK, APO, CYT, CREB, PNK, PIR, PIG, PDG, PEK
PKC delta (Phospho-Thr505)	1.14	CREB, PVE, PTG, PIR, PDG, PGF, PFT, PEK
PKC theta (Phospho-Thr538)	1.86	CREB, PVE, PTG, PTC, PNK, PIR, PIG, PDG, PGF, PEK
PKC zeta (Phospho-Thr560)	1.47	CREB, PVE, PTG, PNK, PIR, PIG, PDG, PGF, PEK
PKD1/PKCmu(Phospho-Ser205)	1.90	CREB, PDG
PKD1/PKCmu(Phospho-Tyr463)	1.24	CREB, PST/PDG
PLC-beta (Phospho-Ser1105)	1.73	AMPK, CYT, PDG, PEK
PLCG1 (Phospho-Tyr783)	1.11	CREB, EGF, PST, PNK, PGF, PEK
PP2A-a (Phospho-Tyr307)	1.37	PKB, CCC, PTG, PST, PMT, PIG, PEK, WNT, MET
PPAR-r (Phospho-Ser112)	2.19	PMT, MET
PTEN (Phospho-Ser380)	1.19	AKT, APO, MET, PKB, APO, PMT, PIG
Pyk2 (Phospho-Tyr402)	1.12	AKT, CYT, MAPK, PST, PDG, PEK

(*Continued*)

Phosphorylation Sites	Ratio	* Signaling pathways
Pyk2 (Phospho-Tyr580)	1.19	PST, PDG, PEK
Pyk2 (Phospho-Tyr881)	2.76	PST, PDG, PEK
Rac1/cdc42 (Phospho-Ser71)	1.13	AKT, PKB, CYT, MET, WNT
RAD52 (Phospho-Tyr104)	1.45	CCC, PST
Raf1 (Phospho-Ser259)	1.97	AKT, APO, CYT, MAPK, CREB, PDGF, EGF, PVE, PTC, PJS, PIR, PGF, PFT, PER, PEK
Raf1 (Phospho-Ser338)	1.14	AKT, APO, CYT, MAPK, CREB, PDGF, EGF, PVE, PTC, PJS, PIR, PGF, PFT, PER, PEK
Raf1 (Phospho-Ser621)	1.18	CREB, PDGF, EGF, PVE, PTC, PJS, PIR, PGF, PFT, PER, PEK
Rb (Phospho-Ser780)	1.71	CCC, PFT
Rb (Phospho-Ser795)	1.25	CCC, PFT
Rb (Phospho-Ser807)	1.74	CCC, PFT
Rb (Phospho-Ser811)	1.21	CCC, PFT
Rho/Rac guanine nucleotide exchange factor2 (Phospho-Ser885)	1.14	CYT, PDGF, EGF, PTG, PMT, PJS, PEK
RSK1/2/3/4 (Phospho-Ser221/227/218/232)	1.16	CREB, PMT, PEK
SAPK/JNK (Phospho-Thr183)	1.13	MAPK, APO, PDGF, EGF, PTG, PNK, PJS, PGF
SEK1/MKK4(Phospho-Ser80)	1.69	MAPK, EGF, PTG, PGF
Shc (Phospho-Tyr427)	1.52	CREB, PDGF, EGF, PVE, PTG, PST, PJS, PIR, PIG, PER
SHP-2 (Phospho-Tyr542)	1.20	PST, PJS, PIG, MET, PGF, PER
SHP-2 (Phospho-Tyr580)	1.23	PST, PJS, PIG, MET, PGF, PER
SLP-76 (Phospho-Tyr128)	1.24	PTC, PST
Smad1 (Phospho-Ser187)	1.14	PTG
Smad2 (Phospho-Ser467)	1.15	PTG
Smad2/3 (Phospho-Thr8)	1.14	CCC, PTG
Smad3 (Phospho-Ser204)	1.46	CCC, PTG
Smad3 (Phospho-Ser213)	1.14	CCC, PTG
Smad3 (Phospho-Ser425)	1.10	CCC, PTG
Smad3 (Phospho-Thr179)	1.68	CCC, PTG
SP1 (Phospho-Thr739)	1.41	PTG
Src (Phospho-Ser75)	1.38	CYT, EGF, PVE, PJS, PDG, PER, PEK
Src (Phospho-Tyr418)	1.38	APO, CYT, MAPK, CYT, EGF, PVE, PST, PJS, PDG, PER, PEK
Src (Phospho-Tyr529)	1.11	APO, CYT, MAPK, CYT, EGF, PVE, PST, PJS, PDG, PER, PEK

(*Continued*)

Phosphorylation Sites	Ratio	* Signaling pathways
SRF (Phospho-Ser77)	1.15	CREB, PDGF, PIR, PEK
SRF (Phospho-Ser99)	1.59	CREB, PDGF, PIR, PEK
STAT1 (Phospho-Ser727)	1.15	APO, ChT, PDGF, EGF, PJS, PEK
STAT1 (Phospho-Tyr701)	1.51	APO, ChT, PDGF, EGF, PST, PJS, PEK
STAT3 (Phospho-Ser727)	1.10	APO, ChT, PDGF, EGF, PGF, PEK
STAT3 (Phospho-Tyr705)	1.35	APO, ChT, PDGF, EGF, PST, PJS, PGF, PEK
STAT5A (Phospho-Ser780)	1.33	APO, ChT, PJS
STAT5A (Phospho-Tyr694)	1.57	APO, ChT, PST, PJS
STAT6 (Phospho-Thr645)	1.97	APO, ChT, PJS
STAT6 (Phospho-Tyr641)	1.23	APO, ChT, PST, PJS
SYK (Phospho-Tyr348)	1.56	PKB, PST, PNK, PER
SYK (Phospho-Tyr525)	1.69	PKB, PST, PNK, PER
Synapsin (Phospho-Ser9)	1.62	CYT
Synucleinalpha (Phospho-Tyr125)	1.21	PST
TAK1 (Phospho-Thr184)	1.15	APO, PTG, PNK, PDG, WNT
Tau (Phospho-Ser214)	1.48	MAPK
Tau (Phospho-Ser356)	1.12	MAPK
Tau (Phospho-Ser396)	1.24	MAPK
Tau (Phospho-Ser422)	1.11	MAPK
Tau (Phospho-Thr181)	1.22	MAPK
Tau (Phospho-Thr205)	1.95	MAPK
TOP2A/DNAtopoisomerase II (Phospho-Ser1106)	1.12	CCC
Tuberin, TSC2 (Phospho-Thr1462)	1.18	PKB, AMPK, PMT, PIG
Tuberin/TSC2 (Phospho-Ser939)	1.12	PKB, AMPK, PMT, PIG
TyrosineHydroxylase (TH) (Phospho-Ser8)	1.14	PDG
VASP (Phospho-Ser157)	1.41	CYT
VEGFR2 (Phospho-Tyr1214)	1.40	NMR, PVE, PST
VEGFR2 (Phospho-Tyr951)	1.37	NMR, PVE, PST
WAVE1 (Phospho-Tyr125)	1.15	CYT, PST
XIAP (Phospho-Ser87)	1.28	PKB, APO, PER
Zap-70 (Phospho-Tyr319)	1.14	AKT, PTC, PST, PNK

*AKT: AKT; APO: Apoptosis; CCC: Cell Cycle Control; CHR: Chromatin Transcription; CYT: Cytoskeletal; MAP: MAPK; MET: Metabolism; NUC: Nuclear Membrane Receptor; PAB: AKT/PKB Signaling; PAM: AMPK; PCR: cAMP-response element binding protein; PDG: PDGF; PEG: EGF; PEK: ERK; PER: ErbB/Her Signaling pathway; PFT: p53 Signaling pathway; PGF: FGF; PGP: G protein coupled receptors; PIG: Insulin Receptor; PIR: IGF-1R; PJS: JAK/STAT Signaling pathway; PMT: mTOR; PNK: NF-κB; PST: Tyrosine; PTC: T-Cell Receptor; PTG: TGF-beta; PVE: VEGF; WNT: WNT Signaling pathway

Supplementary Table S2: The baseline clinical and pathological characteristics of the 203 patients

Parameter	No. of Patients (*n* = 203)		
	MSP positive (*n* = 100)	MSP negative (*n* = 103)	*P* value
**Age (yr)**			
Mean	68.25	71.53	*P* = 0.9517
Median	70	70	
Range	51–84	50–85	
**Preoperative PSA (ng/mL)**			
Median	67.03	16.66	*P*< 0.0001
Range	4.07–190.50	4.10–52	
**Clinical stage**			
T1c	2	54	*P*< 0.0001
T2a	10	37	
T2b	47	10	
T2c	41	2	
**Biopsy Gleason score**			
< 7	34	69	*P*< 0.0001
= 7	15	26	
> 7	51	8	
**Pathologic stage**			
pT2	57	69	*P* = 0.1779
pT3a	23	20	
pT3b	14	13	
pT4	6	1	
**Gleason score**			
< 7	21	77	*P*< 0.0001
=7	19	18	
> 7	60	8	
**Surgical margin**			
Positive	29	3	*P*< 0.0001
Negative	71	100	
**Lymph-node status**			
Positive	56	1	*P*< 0.0001
Negative	44	102	

Supplementary Table S3: Univariate and multivariate models for predicting subsequent biochemical recurrence

Variables	Univariate		Multivariate	
	HR (95% CI)	*P*	HR (95% CI)	*P*
PSA (ng/ml): < 10, 10-20, > 20	1.09 (0.72–1.29)	0.319	-	-
Gleason score: < 7, = 7, > 7	2.26 (1.52–3.23)	0.005	1.95 (1.54–2.21)	0.011
T stage: T2, T3a, T3b, T4	2.79 (2.04–3.41)	0.016	2.54 (1.90–3.23)	0.027
Surgical margin status: negative, positive	3.67 (2.62–4.67)	< 0.001	3.26 (2.52–4.22)	< 0.001
Lymph node status: negative, positive	3.01 (2.49–3.67)	< 0.001	2.83 (1.99–3.53)	0.011
CRMP4 methylation status: negative, positive	7.23 (5.34–9.71)	< 0.001	6.35 (4.64–8.95)	< 0.001
